# DAWN: a framework to identify autism genes and subnetworks using gene expression and genetics

**DOI:** 10.1186/2040-2392-5-22

**Published:** 2014-03-06

**Authors:** Li Liu, Jing Lei, Stephan J Sanders, Arthur Jeremy Willsey, Yan Kou, Abdullah Ercument Cicek, Lambertus Klei, Cong Lu, Xin He, Mingfeng Li, Rebecca A Muhle, Avi Ma’ayan, James P Noonan, Nenad Šestan, Kathryn A McFadden, Matthew W State, Joseph D Buxbaum, Bernie Devlin, Kathryn Roeder

**Affiliations:** 1 Department of Statistics, Carnegie Mellon University, Pittsburgh, PA, USA; 2 Department of Psychiatry, University of California, San Francisco, CA, USA; 3 Department of Genetics, Yale School of Medicine, New Haven, CT, USA; 4 Seaver Autism Center for Research and Treatment, Icahn School of Medicine at Mount Sinai, New York, NY, USA; 5 Department of Pharmacology and Systems Therapeutics and Systems Biology Center New York, Icahn School of Medicine at Mount Sinai, New York, NY, USA; 6 Ray and Stephanie Lane Center for Computational Biology, Carnegie Mellon University, Pittsburgh, PA, USA; 7 Department of Psychiatry, University of Pittsburgh School of Medicine, Pittsburgh, PA, USA; 8 Kavli Institute for Neuroscience, Yale School of Medicine, New Haven, CT, USA; 9 Department of Neurobiology, Yale School of Medicine, New Haven, CT, USA; 10 Child Study Center, Yale School of Medicine, New Haven, CT, USA; 11 Department of Pathology, University of Pittsburgh School of Medicine, Pittsburgh, PA, USA; 12 Program on Neurogenetics, Yale School of Medicine, New Haven, CT, USA; 13 Department of Psychiatry, Yale School of Medicine, New Haven, CT, USA; 14 Departments of Psychiatry, Neuroscience, and Genetics and Genomic Sciences, Friedman Brain Institute and Mindisch Child Health and Development Institute, Icahn School of Medicine at Mount Sinai, New York, NY, USA

**Keywords:** Autism, Risk prediction, Gene discovery, Weighted gene co-expression network analysis, Network, Hidden Markov random field, Neurite extension, Neuronal arborization

## Abstract

**Background:**

*De novo* loss-of-function (dnLoF) mutations are found twofold more often in autism spectrum disorder (ASD) probands than their unaffected siblings. Multiple independent dnLoF mutations in the same gene implicate the gene in risk and hence provide a systematic, albeit arduous, path forward for ASD genetics. It is likely that using additional non-genetic data will enhance the ability to identify ASD genes.

**Methods:**

To accelerate the search for ASD genes, we developed a novel algorithm, DAWN, to model two kinds of data: rare variations from exome sequencing and gene co-expression in the mid-fetal prefrontal and motor-somatosensory neocortex, a critical nexus for risk. The algorithm casts the ensemble data as a hidden Markov random field in which the graph structure is determined by gene co-expression and it combines these interrelationships with node-specific observations, namely gene identity, expression, genetic data and the estimated effect on risk.

**Results:**

Using currently available genetic data and a specific developmental time period for gene co-expression, DAWN identified 127 genes that plausibly affect risk, and a set of likely ASD subnetworks. Validation experiments making use of published targeted resequencing results demonstrate its efficacy in reliably predicting ASD genes. DAWN also successfully predicts known ASD genes, not included in the genetic data used to create the model.

**Conclusions:**

Validation studies demonstrate that DAWN is effective in predicting ASD genes and subnetworks by leveraging genetic and gene expression data. The findings reported here implicate neurite extension and neuronal arborization as risks for ASD. Using DAWN on emerging ASD sequence data and gene expression data from other brain regions and tissues would likely identify novel ASD genes. DAWN can also be used for other complex disorders to identify genes and subnetworks in those disorders.

## Background

That genetic variation affects the risk for autism spectrum disorders (ASDs) has been known for decades, yet only recently has the complexity of its architecture come into focus [1]. During the past few years a series of studies has been published, some analyzing copy number variants [2,3], others rare sequence variants [4-9], and still others common variants [10,11], whose data can only be explained if many genes are involved in the risk for ASD. Our recent work estimates this number to be about 1,000 [1,9,12], a remarkably high fraction of the known genes in the genome. To date, analysis of 1,043 ASD trios has identified a handful of the genes involved in the ASD risk. Extrapolating from these data would require exome analysis of tens of thousands of families to identify even half of the risk genes, an infeasible short-term goal with regard to sample collection and funding. Therefore there is an urgent need to advance ASD gene discovery through the integration of complementary biologically relevant datasets.

The complexity of the ASD genetic architecture raises challenges, but we anticipate there will be a discoverable organization to these genes that will pave the way for deep insights into genetics and neurobiology. Support for this conjecture comes from recent analyses [13-15]. A recent paper [14] has laid the foundation for these insights in two ways: by identifying brain gene expression networks as meaningful for organization and interrelationships of ASD genes; and by identifying the region and developmental periods in which these genes tend to coalesce to confer risk of ASD, specifically the mid-fetal prefrontal and motor-somatosensory neocortex (PFC-MSC). We reasoned that if this region were a critical nexus for the expression of ASD genes, it would be the perfect place to hunt for novel ASD genes. Thus we take the results from [14] further by integrating two key data sets, BrainSpan gene expression [16] and results from analysis of rare sequence variation [12], to identify genes and subnetworks in the mid-fetal PFC-MSC that likely underlie ASD risk.

**Figure 1:**
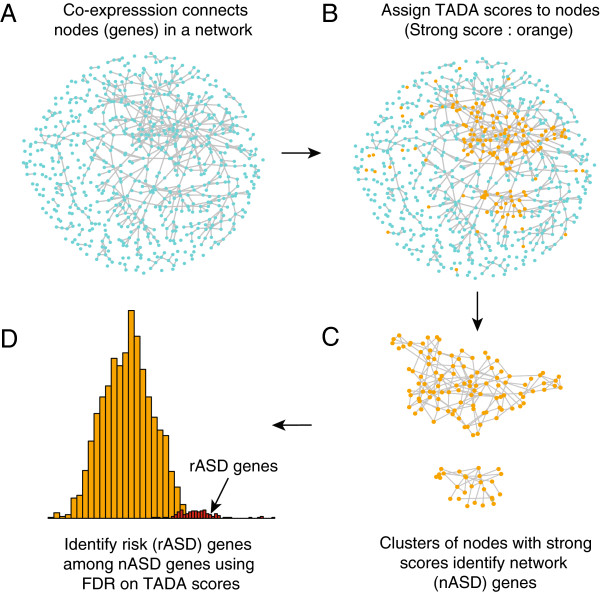
**The DAWN algorithm.(A)** Each node in the network represents a gene and each edge represents pairs of genes with strong co-expression (absolute correlation *r*>0.7). **(B)** Orange nodes indicate genes with strong genetic scores from the TADA test. **(C)** Hot spots (i.e., clusters of strong scores) are classified as nASD genes in the screening stage of the algorithm; cool spots (i.e. strong scores in isolation) are not.**(D)** In the final cleaning step, the nASD list is further refined to reveal the rASD gene list. This step uses the TADA scores and features of the network to compute the false discovery rate of each gene. FDR, false discovery rate; nASD, network autism spectrum disorder; rASD, risk autism spectrum disorder; TADA, transmission and *de novo* association.

To implicate genes in risk (predicted risk or rASD genes) we have developed an algorithm named DAWN (for Detecting Association With Networks, Figure 1). Building on the logic that ASD genes cluster within a co-expression network [14,15], the algorithm identifies ‘hot spots’ within this co-expression network at which multiple genes with evidence of ASD association from the exome data cluster together. For these hot spots DAWN uses the evidence from neighboring genes to reinforce the ASD signal, while in ‘cooler’ regions the absence of neighboring genes with evidence of ASD association downgrades the signal. By modeling these data, DAWN identified 127 rASD genes (Table 1), many of which are novel. By analyzing independently generated association data [17] for a subset of these rASD genes we validated DAWN by demonstrating its ability to delineate which genes will yield new *de novo* mutations and which will not. Importantly these results provide a framework for targeted resequencing of new samples to demonstrate involvement in ASD risk definitively and for neurobiological assessment of gene and subnetwork function. Moreover, this approach could be applied to other gene expression data in relevant tissues to identify additional subnetworks of ASD risk genes.

**Table 1:** List of genes predicted to affect risk for ASD (rASD genes)

		**Range of FDR** ***q*-values**^**∗**^	
**Number of dnLoF mutations**	**0-0.0025**	**0.0025-0.025**	**0.025-0.05**
>1	*CUL3*, *DYRK1A*, ^d^*GRIN2B*, ^a,d^		
	*POGZ*, *SCN2A*, ^a^*TBR1*^a,d^		
1	*ADNP*, ^d^*CBX4*, *CDC42BPB*,	*ARID1B*, ^d^*ATP1B1*, *BCL11A*,	*RIMS1*^e^
	*COL25A1*, *DIP2C*, *DDX3X*,	*CSTF2T*, *FOXP1*, ^b^*ITGA5*,	
	*LMTK3*, *MED13L*, *NFIA*,	*L1CAM*, ^c^*NCKAP1*, *MBD5*,^a^	
	*RAB2A*, *PHF2*, *RNF38*,	*PCOLCE*, *SCP2*, *SHANK2*, ^a^	
	*PPM1D*, *PRPF39*, *SETBP1*,	*SPAST*, *SMARCC2*, *TCF3*,	
	*TROVE2*, *UBR3*, *ZMYM2*	*UNC80*, *VCP*	
0	*BANK1*, *C1orf95*, *ELOVL1*,	*AGK*, *ARSK*, *ATRN*, *BBS10*, ^b^	*ACTL6A*, *ANKS1B*, *ASB8*,
	*FCAR*, *LMCD1*, *SMC3*,	*BEND7*, *C2CD3*, *CD34*,	*BAHCC1*, *C1orf43*,
	*PRIM2*, *PTEN*, ^c,d^*SERINC5*,	*CHMP2B*, *CLDN11*, *CNOT1*,	*CASD1*, *CDC42EP4*, *DUSP14*,
	*SMAP1*, *TNC*,	*CRY1*, *DCAF11*, *DHX29*,	*HCFC2*, *HIST1H3D*, *LYSMD3*,
	*ZNF175*, *ZNF33A*	*DYNC1I2*, *EIF3G*, *F3*, *FBXL5*,	*MARK4*, *NAV2*, *PAMR1*,
		*GDPD4*, *GMNN*, *HIST1H4B*,	*PCNX*, *PSMG2*, *RSU1*,
		*KIAA1468*, *ITGB3BP*, *MAPK4*,	*SMPD3*, *SPRY1*, *TNPO3*,
		*MCM5*, *MAPT*, *MARCO*,	*VASH1*, *ZNF410*
		*METTL14*, *MRPS26*, *MRPL44*,	
		*MUDENG*, *NCOR1*, *NDUFB5*,	
		*NIF3L1*, *NR2F1*, *OR2AK2*,	
		*PCIF1*, *PDLIM1*, *RAD21*,	
		*RAD51AP1*, *RBBP9*, *REXO1*,	
		*RNF168*, *SCD*, *SLC22A15*,	
		*SMG7*, *SPAG17*, *STXBP1*, ^c^	
		*TBL1XR1*, ^d^*TSR1*, *ZFAND2A*	

^*^ASD genes are displayed by range of False Discovery Rate (FDR) q-value; the 3 columns correspond to genes significant with a genome-wide correction at levels.0025,.025, and.05, respectively. Genes with FDR <.05, but validation score less than.90 were not included. ^a^gene with strong prior support for affecting risk for ASD; ^b^gene with modest prior support for affecting risk for ASD; ^c^syndromic gene; ^d^gene with a *de novo* loss-of-function mutation in the [17] study; ^e^RIMS1 has one *de novo* loss-of-function mutation, netscore = 107 (95th percentile), and FDR of.077 exceeding the cutoff.

## Methods

### Gene expression and co-expression

The data analyzed were produced as previously described [16] and based on the same quality control and quantile normalization. After total RNA was extracted from tissue samples, gene expression was assessed using the Affymetrix GeneChip Human Exon 1.0 ST Array platform (Platform GPL5175), yielding high-quality comprehensive data. The data were downloaded from the National Center for Biotechnology Information Gene Expression Omnibus (GEO accession number [GEO:GSE25219]). Expression data from the core probe set were used in co-expression analysis of most genes. For genes *CHD8*, *FLG*, *FREM3*, *FRG2C*, *LMTK3*, *THSD7A*, *UBN2* and *ZNF594*, however, data from the extended probe set were utilized. We utilized measurements from PFC-MSC, analyzing 14,651 unique transcripts [16]. To investigate mid-fetal development we targeted post-conception weeks 10–24, which covers time periods 3–6 as defined previously [16]. In our analysis, we used two overlapping windows: periods 3–5 (post-conception weeks 10–19) and 4–6 (post-conception weeks 13–24) with 10 and 14 brains available, respectively.

Gene co-expression was measured by the Pearson correlation *r* between pairs of genes. To obtain the co-expression between a pair of genes *X* and *Y*, multiple observations of the joint expression of *X* and *Y* are essential. These replicates were obtained in two ways, by measurements of *X* and *Y* from different regions of the same portion of the brain, and from the same region in different brains. For periods 3–5 and 4–6 there were 107 and 140 replicates of expression per gene, respectively (Additional file 1: Table S1).

### Gene networks

Gene networks were inferred from the pairwise correlation matrices using the software package Weighted Gene Co-expression Network Analysis (WGCNA) [18,19]. A similarity matrix was calculated from the absolute correlation of gene expression (*r*) raised to a power. For each pair of genes, a topological overlap measure was calculated based on the adjacency matrix. From the implied dissimilarity between genes, average linkage hierarchical clustering was used to construct the dendrogram. Modules were chosen using dynamic cutting of the branches of the resulting clustering tree. We set the minimum module size to 30 genes and the minimum height for merging modules at 0.15. Closely related modules can be merged using the adjacency of eigengenes (i.e., the first eigenvectors of the expression matrix for a module). To capture salient features of the gene co-expression network fully, modules were built independently for each time span (3–5 and 4–6), and within each period of development modules were chosen using two different choices of the power parameter (1 and 6); see Additional file 2: Figure S1, Additional file 3: Table S2 and Additional file 4: Table S3 for details. The first step of the DAWN algorithm (Figure 1) involves evaluating these four representations of the gene expression data. Multiple representations are necessary because a single partition of genes into highly co-expressed modules fails to capture the full neighborhood of all genes; using multiple sets of modules avoids missing signals from risk genes that are on the boundary between two modules. The goal here is for every gene to have its nearest neighbors included in a common module for at least one partition of the genes.

Within each module we clustered highly correlated genes to create multi-gene nodes. For these analyses the tree was cut at height |*r*|=0.75 to yield the genes in a multi-gene node. Once the complete set of nodes was defined (both single-gene and multi-gene), a network was constructed by connecting nodes that are correlated at the next level of strength (|*r*|>0.7). We chose a threshold of *r*=0.7 for the network because it is a widely used threshold in the literature and it provided the desired network density. Specifically, we found that *r*=0.6 produced a very dense network and *r*=0.8 a very sparse network, each unsuitable for the proposed analysis. Our motivation for pre-clustering highly correlated genes as multi-gene nodes was to create a sparse network that was not dominated by local subsets of highly connected genes. By grouping these subsets of genes into multi-gene nodes, the broader pattern of network connections becomes more apparent. Naturally to work within the algorithm as a whole, the threshold for multi-gene clusters must be greater than 0.7. For *r*=0.8 only a small number of genes would be clustered, however, and therefore.75 was chosen as a compromise between theseextremes.

### Genetic data from whole-exome sequencing studies

Transmission and *de novo* association (TADA) scores [12] (Additional file 5: Table S4) were calculated from the following data: all reported *de novo* mutations from 932 ASD families consisting of trios of affected offspring and two parents from four studies [4,6,8,9]; transmitted rare variants from 641 of these families from two studies [4,9]; and case-control data from the ARRA Autism Sequencing Consortium, consisting of 935 ASD subjects and 870 controls [20]. In addition we included two *de novo* loss-of-function (dnLoF) mutations obtained from a set of 44 trios [5] and 56 trios [14]. For a complete list of *de novo* variants utilized, see [14]. Each missense mutation was classified into a category of damage to the protein based on its predicted effect on the coding sequence using PolyPhen2 [21]. Loss-of-function (LoF) and ‘probably damaging’ missense variants were analyzed by TADA, both of which showed enrichment in probands for these data. In addition to finding strong statistical support for a few novel ASD risk genes [12], TADA found significant enrichment of genes with small *P* values compared with random expectation, indicating there are more genes affecting risk for ASD yet to be discovered, even from these genetic data.

The TADA *P* values were converted to *Z*-scores using the standard normal probability integral:

$$Z=\Phi^{-1}(1-P)$$

where *Φ* is the cumulative distribution function of standard normal distribution. Provided a gene is not associated with ASD, it follows without further assumption that the *Z*-score is standard normally distributed. When a gene is a risk gene, the *Z*-score approximately follows a normal distribution with mean *μ*>0. A *Z*-score is associated with each node. For multi-gene nodes this is the minimum *P* value of all genes in the node.

### The DAWN algorithm

From a statistical perspective, DAWN is based on the ‘screen and clean’ principle [22]: first screen the data to find all potential signals (network ASD or nASD genes), and then using more stringent criteria, clean the list so that it includes only those signals that meet more traditional criteria for significance (rASD genes). This basic strategy has been shown to increase power and yet control error rates in a similar high-dimensional setting [22].

#### Screening stage

DAWN relies on a hidden Markov random field (HMRF) to identify clusters of possible risk genes embedded in the entire expression network (Figure 1, Additional file 6: Figure S2 and Additional file 7: Text S1). The true state of each node (rASD risk or not) is hidden, but the TADA score associated with gene node can be observed. Clusters of nodes with high TADA scores suggests that these nodes are likely associated with risk. The HMRF network algorithm works as follows: (1) genes are organized into highly correlated modules based on gene expression using WGCNA, (2) the adjacency matrix defines a network including edges between genes with absolute correlation exceeding a fixed threshold, (3) this model examines the initial signals provided by the node *Z*-scores to determine if high scores tend to be clustered in the network and (4) the fitted model then infers the missing label for a node, namely whether it is related to ASD risk or not. This label is determined based on the *Z*-score of the node and whether or not the node has many neighbors with large *Z*-scores. By using a number of computational approximations, including the iterative conditional mode, the model parameters can be estimated efficiently. Consequently we can estimate the probability a node is associated with ASD risk. For related literature, see [23,24]. We use a posterior probability of 0.5 to identify nodes potentially associated with risk and call the genes in these nodes network ASD (nASD) genes.

As described earlier, tightly clustered genes are collapsed into multi-gene nodes. The adjacency matrix entries for these nodes are defined based on the average linkage between nodes. Each multi-gene node is assigned a node score defined by the minimum *P* value of all genes within the cluster. Finally, the HMRF analysis follows as for single gene nodes. In this way, the HMRF algorithm can be applied to a much smaller set of nodes with an adjacency matrix that is far less densely connected. Based on results from simulations and data analysis, it appears that the HMRF approach is more powerful at detecting clusters of risk nodes when multi-gene nodes are incorporated into the algorithm.

#### Cleaning stage

After running the HMRF model, the goal at this step is to winnow the nASD list down to a smaller set of genes that are highly likely to affect risk on the basis of the genetic evidence using a false discovery rate (FDR) procedure [25]. We call these probable risk (rASD) genes. To maximize power to discover rASD genes in subnetworks dense for genes affecting risk, we use a stratified analysis. Each large multi-gene node defines a stratum (more than ten genes), and we fit a Gaussian mixture model to the distribution of TADA *Z*-scores to estimate the fraction of risk genes present in the multi-gene node [26]. The larger this fraction is estimated to be, the larger the number of genes determined to be rASD genes. Thus this FDR procedure garners power by exploiting the heterogeneity inherent across multi-gene nodes and modules, while still controlling the error rate. Then, for all remaining nASD genes, which includes small multi-gene nodes, the distribution of TADA test statistics is evaluated by fitting the mixture model to the entire set of statistics (Additional file 8: Figure S3). The model is described in detail in Additional file 7: Text S1.

The DAWN analysis is performed for power 1 and power 6 modules and for periods 3–5 and 4–6 PFC-MSC. Thus there are four representations of the gene expression network. To select a unique set of rASD genes we use the minimum FDR across four representations.

### Permutation experiments

To evaluate DAWN we performed two permutation experiments. Each sought to illuminate DAWN’s performance by diluting the signal for association in two ways: (I) by separating small *P* values from risk genes and (II) by moving risk genes from clusters of genes with small *P* values. All of the permutation experiments were performed at the node level. Hence single gene nodes and multi-gene nodes were treated interchangeably in what follows.

#### Experiment I: diluting signals

1. Randomly select a proportion *l* of nodes that have *P* values less than or equal to 0.1. The proportion *l* is set to be equal to 0.2, 0.4, 0.6, 0.8 or 1.

2. Randomly select the same number of nodes that have *P* values greater than 0.1. Permute the *P* values of selected nodes with the nodes selected in step 1.

3. Run the HMRF approach with the permuted data and estimate the parameters of the model. Record the number of genes identified that have at least one dnLoF variant.

4. Repeat steps 1–3 20 times for each *l*.

#### Experiment II: diluting the clustering of signals in the network

Replace Step 2 above with the following: 

•Randomly select the same number of nodes that have *P* values greater than 0.1. Permute the selected nodes (i.e., switch both the *P* value and the gene labels associated with the pair of nodes). With increasing dilution, this effectively removes the correlated nature of the signal.

### Network score

To summarize gene *i*’s position within the network, a network score was calculated as:

$$S_{i}=\underset{j\neq i}{\sum }|r_{\text{ij}}|\times z_{j}$$

in which both variables are given hard thresholds (0 if correlation |*r*_*ij*_|<0.7 or if *Z*-score *z*_*j*_<1.2). The *Z*-score is obtained from the TADA *P* value.

### Connectivity

To evaluate the connectivity of the rASD gene list we performed a permutation test. All genes expressed in the brain that fell within a module and had exome data were identified: 10,223 genes matched these criteria including all 127 rASD genes. The genes were sorted by mutability (based on size and GC content). Random lists of 127 genes were sampled repeatedly, with the constraint that they be approximately equal in mutability to the original list. We compared the mean connectivity of each list of 127 to the true rASD list to obtain a *P* value for connectivity.

### *De novo* probability model

We estimated the probability that a true ASD gene has at least one dnLoF mutation in a sample of 2,500 trios by extrapolating from available trios. In a sample of 1,043 trios, 143 *de novo* LoF mutations were observed, involving 130 unique genes, with 9 genes incurring multiple events and 121 incurring single events [14]. Extrapolating this process to 2,500 trios we expect about 342 *de novo* LoF mutations, involving about 311 unique genes, with about 13 genes incurring multiple events and 298 incurring single events. Based on previous analysis, we anticipate about 50% of the single-mutation genes and most of the multi-mutation genes are true ASD genes [14]. Consequently, we predict approximately 162 (or slightly fewer) true ASD genes will have at least one *de novo* LoF mutation in a sample involving 2,500 trios. Assuming there at least 1,000 true ASD genes [12], each ASD gene, *a priori*, has approximately a 15–16% chance of having a dnLoF mutation in a sample of 2,500 trios.

### Protein-protein interactions

A literature-based protein-protein interaction (PPI) network was constructed by combining interactions from the following databases: BioGRID [27], HPRD [28], MINT [29], IntAct [30], KEA [31], KEGG [32], SNAVI [33] and MIPS [34]. Only interactions from publications that reported ten or fewer interactions were retained. After combining the binary interactions from these databases by converting gene IDs to EntreZ Gene Symbols the biggest connected component was used for further analysis. rASD genes were seeded in this network and Dijkstra’s shortest path algorithm [35] was used to extract a subnetwork that connected the seed genes using a path length of three (one intermediate and two links) [36]. The natural clustering of the obtained subnetwork was created using the organic layout of the graphic software yEd [37]. The relative local clusters were manually identified.

### Functional enrichment analysis with Enrichr

Seed rASD genes from the identified clusters of the PPI network, together with the intermediates from each cluster, were fed into the online gene enrichment analysis tool Enrichr [38]. Enrichr has 36 gene set libraries and performs gene set enrichment analyses using Fisher’s exact test (FET) with Benjamini Hochberg corrections [25]. We focused our enrichment analysis on functional annotation from gene set libraries created from the gene ontology (GO) [39] biological process (BP) tree, the gene ontology molecular function (MF) tree and the mouse genome information (MGI) molecular phenotype (MP) browser ontology tree [40]. Enrichr [38] and its accompanying databases are online [41].

## Results and discussion

### Discovering network and risk genes using DAWN

To search for clusters of possible risk genes embedded in the entire expression network, DAWN models the interrelationships amongst nodes of a network, in terms of risk status, and combines such interrelationships with node-specific genetic signals for association (Figure 1). As part of this process, DAWN assigns nodes a posterior probability of being part of an ASD subnetwork. Genes are defined as nASD genes if their posterior probability from the hidden Markov analysis crosses the threshold of 0.5. This results in 2,323 genes being classified as nASD genes (Additional file 5: Table S4).

To illustrate the efficacy of DAWN for this step, we computed the network score for each gene, which quantifies the strength of the genetic signal from the neighboring genes in the network. The distribution of these scores demonstrates that nASD genes are indeed significantly more connected to other genes potentially affecting risk when compared to genes not in this set (Methods, Additional file 3: Table S2; and Additional file 8: Figure S3; Wilcoxon rank test, *P*<10^−16^).

Once genes are separated into the nASD and non-nASD groups (the screening step of the screen-and-clean algorithm), DAWN performed a further evaluation of the genetic data with the goal of finding the subset of nASD genes with compelling evidence for ASD risk (cleaning step). The cleaning step has two levels, an FDR procedure tailored to the network structure, the genetic score, and an evaluation of sensitivity of the FDR results to network structure. After performing the FDR procedure, 146 genes have *q*≤0.05 (Additional file 5: Table S4).

To examine the robustness of these predictions to network structure, we performed a cross-validation experiment in which we iteratively removed a fraction of the genetic signals and re-evaluated the prediction of risk genes. For each iteration, we randomly removed the genetic signal from 10% of the rASD genes and then reran the DAWN algorithm to determine which of the remaining rASD genes were identified as predicted risk genes by the algorithm. A ‘validation score’, specifically the fraction of iterations for which the gene is included in the updated list, showed that most of the rASD genes are robust to the set of genetic signals present (Additional file 5: Table S4), with 86% achieving a validation score of 90% or higher. However, for the other 14% of genes originally predicted to affect risk, the results depended on a small number of neighboring genes and were sensitive to their removal. These genes were excluded from the final rASD list because their association was judged to be non-robust, leaving 127 genes that were predicted to affect risk (Table 1).

**Figure 2:**
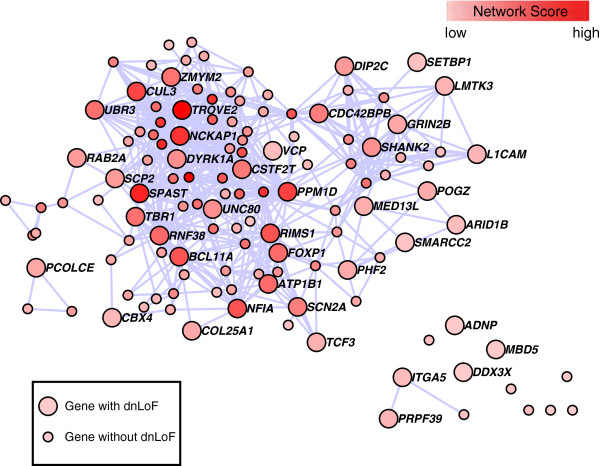
**Network of risk ASD (rASD) genes.** These genes met the false discovery rate threshold of.05. The intensity of the red reflects the magnitude of the netscore based on TADA statistics from neighboring genes. Large nodes depict genes that have at least one dnLoF mutation. *CUL3*, *DYRK1A*, *GRIN2B*, *POGZ*, *SCN2A* and *TBR1* are genes with multiple dnLoF mutations. Edges connect genes with absolute correlation of.7 or greater based on periods 3–5 or 4–6. dnLoF, *de novo* loss of function; rASD, risk autism spectrum disorder; TADA, transmission and *de novo* association.

The rASD genes had striking co-expression (Figure 2), significantly different from a random set of brain-expressed genes with similar attributes (*P*<0.000001; Methods). On the other hand, the network scores for the rASD list are not significantly greater than other nASD genes (Additional file 8: Figure S3), implying that inclusion in the rASD list requires a high network score coupled with at least a moderate level of genetic evidence for association (Additional file 9: Figure S4).

### Evaluating how well DAWN works

To assess DAWN’s performance we focused on the number of genes identified by the algorithm that have at least one dnLoF mutation in sequenced probands, since the 127 rASD genes are highly enriched for such mutations (Table 1) and over 50% of such genes are independently predicted to be ASD risk genes [9]. If DAWN were using both the network (gene expression) and association (exome) results effectively, we would expect that diluting either of these features would diminish its ability to detect these dnLoF genes. To evaluate this question we conducted two permutation experiments, either diluting the association by separating likely risk genes from their small *P* values or diluting the network by breaking up clusters of genes with small *P* values and randomly distributing them throughout the network (Methods). The degree of dilution varied from 0% to 100%.

For both dilution experiments, we found that the performance (i.e. the number of dnLoF genes identified) decreased in almost direct proportion to the degree of dilution (Additional file 10: Figure S5). This result demonstrates that DAWN is sensitive to both the ASD association signal (exome) and the location of a gene within the network (gene co-expression) in its selection of rASD genes (Figure 1, Additional file 6: Figure S2).

Conversely, DAWN does not require overwhelming prior evidence of association to identify rASD genes. To test this we considered six rASD genes that have multiple dnLoF mutations leading to a very low prior *P* value calculated by TADA (Table 1). By downgrading these six genes to single dnLoF mutations, the recalculated TADA *P* value was increased by several orders of magnitude, as expected. Yet, on rerunning DAWN with these higher *P* values, all six of these genes were still predicted to affect risk, clearly indicating that they were pulled into the rASD gene sets based on the strength of their connections to neighboring genes with evidence of ASD risk.

DAWN does require genes to be connected in the adjacency matrix. Yet expression modules can create artificial boundaries that separate some gene clusters. For this reason we used four different modular representations of the gene expression network in DAWN. When genes are selected as rASD genes, closer inspection reveals that they typically share a module with most (or all) of their top 20 nearest neighbors for all representations. Some genes, however, appear as rASD genes for only one representation and are often separated from several of their nearest neighbors for the other representations. The rASD gene *SHANK2* provides a good example. In periods 4–6 PFC-MSC, *SHANK2* was in a module with four other rASD genes for one representation of the network data and was identified as an rASD gene; for the other representation, however, it was in a module with no other connecting rASD genes and was not detected. For this reason we believe it is essential to use multiple modular representations of the gene co-expression network.

### Validation of rASD genes

#### Analysis of resequencing experiment

On average half of all dnLoF mutations in ASD probands correspond to true ASD genes [9], hence one way to evaluate the rASD list is to compare it to a list of genes with dnLoF mutations identified based on sequencing of independent ASD families. Calculations based on empirical *de novo* rates and a new set of 2,448 ASD trios show the chance of seeing a dnLoF mutation in a particular ASD gene is about 15%, although this varies depending on gene size and relative risk. Consequently, most true ASD genes will have no dnLoF mutations, even in this relatively large study, and thus direct validation of individual genes in the rASD list is infeasible. Nonetheless they can be evaluated as a group for enrichment of dnLoF mutations in additional trios. Under this scenario a compelling experiment has already been performed, namely targeted sequencing of a sample of 2,448 trios with molecular inversion probe sequencing (MIPS) of 44 carefully selected ASD candidate genes (henceforth known as the MIPS experiment [17]). Ten of these 44 candidate genes are also on the rASD list, thus they can be evaluated to determine if they had an unusually high number of dnLoF mutations in the MIPS experiment.

In the MIPS experiment, eight genes incur at least one additional dnLoF event and six of these are on the rASD list (*ADNP*, *ARID1B*, *DYRK1A*, *PTEN*, *TBL1XR* and *TBR1*), demonstrating significant enrichment (*P*=0.0007). Of the two genes incurring additional dnLoF but missing from the rASD list, *CHD8* is an obvious ASD gene [17], but its expression levels were derived from a less reliable extended probe set, while the other analyzed genes were present on the core probe set of the BrainSpan exon array data. *CHD8*’s expression is not tightly correlated with that from other genes, hence it is excluded from the nASD gene set. The other gene, *CTNNB1*, is an nASD gene, but it has a TADA *P* value of 0.36 *a priori* and hence DAWN does not predict it as a risk gene. Of the four rASD genes that did not sustain dnLoF mutations in this study, three are known ASD genes (*CUL3*, *FOXP1* and *MBD5*) and one is a syndromic gene for which ASD is sometimes a comorbid outcome (*SETBP1*).

**Figure 3:**
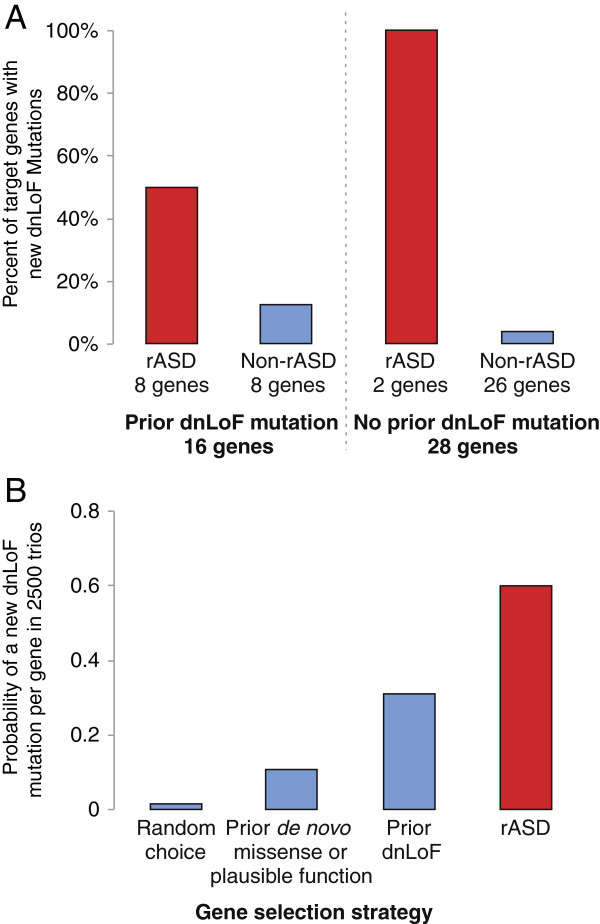
**Analysis of MIPS validation experiment.(A)** First 44 genes with prior *de novo* mutations were sequenced for 2,448 additional trios. These genes were cross-classified by whether or not they had a prior dnLoF mutation, and whether or not they were on the DAWN rASD list (yes: red, no: blue). For each category, the percentage of genes that had a dnLoF mutation in the new trios is depicted.**(B)** For a given gene, the probability of observing a dnLoF mutation in 2,500 probands varies. This probability is compared for four types of genes: a randomly chosen gene and three classifications of the genes from the MIPS experiment including: (i) all 44 genes, (ii) those 16 genes with a prior dnLoF mutation and (iii) those 10 genes on the rASD list. dnLoF, *de novo* loss of function; rASD, risk autism spectrum disorder.

In this experiment DAWN was able to distinguish the genes that will accumulate new dnLoF mutations better than any existing methods (Figure 3, Additional file 11: Table S5). DAWN identified two rASD genes for which no dnLoF mutations had previously been observed; in the MIPS experiment new dnLoF mutations were identified for both of these (100% success rate) compared with the 26 genes with no previous dnLoF mutation that were not on the rASD gene list (4% success rate, FET *P*=.008, odds ratio =*∞*). DAWN also outperformed the other methods for genes with previous dnLoF mutations: new dnLoF mutations were identified for four out of eight rASD genes (50% success rate) compared with one out of eight that were not on the rASD gene list (13% success rate, FET *P*=0.14, odds ratio 6.16).

From the results of the original experiment, we conclude that many of the 44 genes selected for the MIPS experiment are likely ASD genes because the rate of dnLoF mutations is more than would be expected even if all 44 were true ASD genes. Still DAWN appears to do markedly better (Figure 3B). We conjecture its better performance is largely due to identifying ASD genes with higher relative risk, compared to the average ASD gene. Genes with a larger relative risk are more likely to have dnLoF mutations *a priori*[12], an expectation also supported by the MIPS experiment.

#### Previously identified autism spectrum disorder genes and probable risk genes

Of the rASD genes nominated (Table 1), six have been implicated for ASD risk on the basis of multiple dnLoF events in exome sequencing studies (*CUL3*, *DYRK1A*, *GRIN2B*, *POGZ*, *SCN2A* and *TBR1*). Seven others have been identified as ASD genes on the basis of published research [42] (three syndromic: *L1CAM*, *PTEN* and *STXBP1*; two with strong support from copy number and sequence studies: *MBD5* and *SHANK2*; and two with equivocal evidence: *BBS10* and *FOXP1*). This demonstrates significant enrichment (FET *P*<10^−6^) for nominal ASD genes in the rASD list.

Within the rASD set (Table 1) are 36 genes containing a single dnLoF mutation known from prior exome sequencing studies [4-6,8,9,14], demonstrating significant enrichment (FET *P*<10^−16^) compared to the full list of 116 such genes with quality expression data for the mid-fetal PFC-MSC. Moreover three more rASD genes were found to have a dnLoF mutation by the MIPS experiment (*TBL1XR1*, *ARID1B* and *ADNP*). These results are of note because of the expectation that roughly 50% of these genes are involved in risk [9] and DAWN does a better than expected job at identifying these 50%.

#### Functional coherence

**Figure 4:**
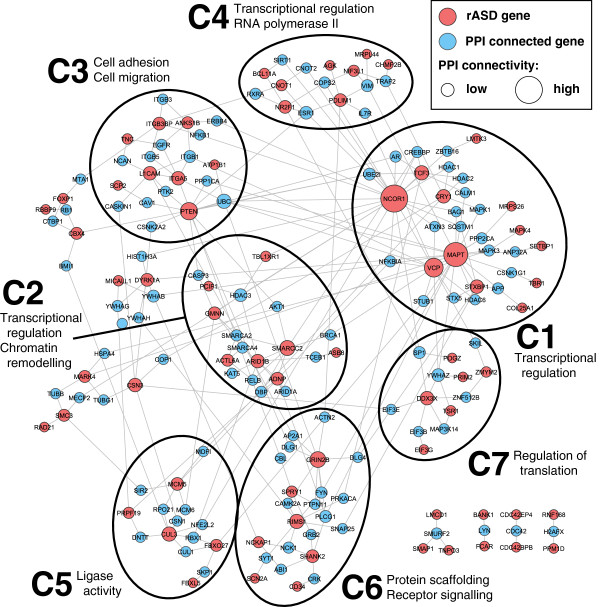
**Clustering by enrichment and protein-protein interaction (PPI).** The rASD genes are seeded into the PPI network presented in [6], represented by red nodes, with size proportional to the number of connections. The blue nodes are immediate intermediate proteins [36]. The network was clustered using organic clustering methods implemented in yEd [44] rASD, risk autism spectrum disorder.

Next we reasoned that if the rASD list were meaningful, it should be enriched for biologically meaningful, ASD-relevant processes. We focused on PPI networks, which are independent of the co-expression networks we analyzed but have the expectation that interacting genes will have correlated expression. In addition to forming a highly significant network of interacting genes (Additional file 12: Figure S6), the rASD genes in the PPI network fall into several natural clusters (Figure 4). Clusters C1, C2 and C4, accounting for a large proportion of the genes, share related functional categories. Specifically, these three clusters are involved in transcriptional regulation (see the GO BP and GO MF categories in Additional file 13: Figure S7). Cluster C2 is additionally enriched for chromatin remodeling terms in GO BP, while cluster C4 is enriched for RNA polymerase II-related categories in GO MF. Additionally Cluster C7 relates to regulation of translation as seen in both GO BP and GO MF. Together these results show that dysregulation of gene expression and coordinated co-expression is a key risk factor for ASD and they further suggest dysregulation has an effect early in development. Dysregulation of coordinated gene expression is consistent with a wide range of ASD studies [43].

Among other processes, clusters C3, C5 and C6 map onto neuronal migration and function, both thought to be involved in ASD risk [45,46]. Cluster C3 is enriched for GO BP categories involved in cell adhesion and cell migration and for abnormalities in cell migration in MGI MP. This cluster shows strong enrichment for the KEGG category of focal adhesion (HSA04510). Cluster C5 is enriched for GO MF categories around ligase activity, including ubiquitin-protein ligase activity. This cluster shows a similar enrichment in KEGG, for ubiquitin-mediated proteolysis (HSA04120), previously implicated in neuronal function and ASD risk [47]. Cluster C6 is enriched for GO MF categories around protein scaffolding and receptor signaling. This cluster is also associated with several important MGI phenotypes, including lethality and abnormalities in neuronal morphology, synaptic transmission and plasticity, and learning and memory.

### Subnetworks

**Figure 5:**
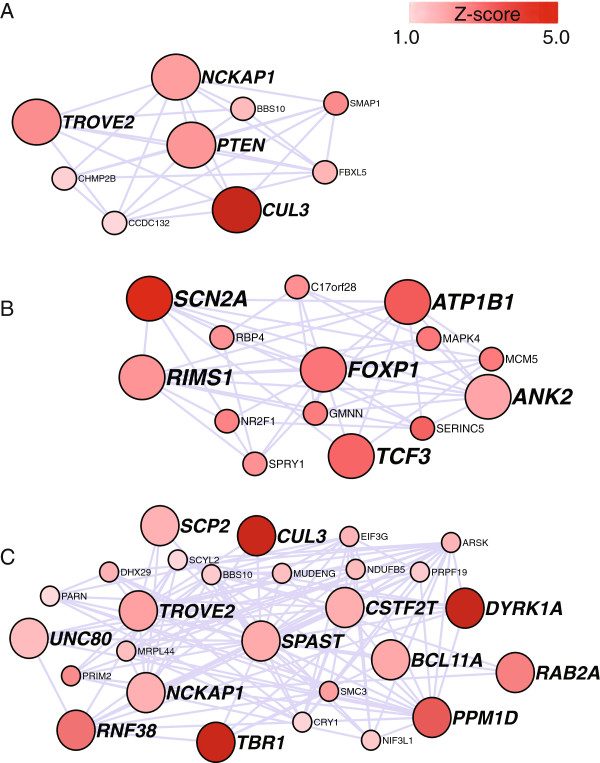
**Gene subnetworks for the*PTEN*,*FOXP1* and*SPAST* genes.** Figure shows all rASD genes with absolute correlation of.7 or better with **(A)***PTEN*, **(B)***FOXP1* and **(C)***SPAST*. Intensity of red color reflects the magnitude of the *Z*-score from the TADA statistic. Large nodes with labels depict genes that have at least one dnLoF mutation recorded in the current literature (except *PTEN*). *ANK2*, *CUL3*, *DYRK1A*, *SCN2A* and *TBR1* are genes with multiple dnLoF mutations. *ANK2* is included along with rASD genes because it has two dnLoF mutations.

The set of nASD genes, and especially the rASD genes, define subnetworks of co-expression, which can be used to focus further neurobiological research (Additional file 5: Table S4). We highlight four subnetworks for illustration: one centered on *PTEN* (Figure 5A), which is a syndromic gene in which mutations are known to increase ASD risk; one centered on *FOXP1* (Figure 5B), encoding forkhead box P1, for which there is some *a priori* evidence for involvement in ASD risk [48]; one centered on *SPAST* (Figure 5C), encoding spastin, which has no known involvement in ASD risk; and one centered on *VRK1*, encoding vaccinia related kinase 1, an nASD gene that has a very high network score and which did not pass the threshold for the rASD list (Additional file 14: Figure S8).

Although *PTEN* is a known ASD gene, existing whole exome sequencing data do not yet provide compelling evidence for its involvement in risk (uncorrected TADA *P*=0.0025, insignificant after accounting for testing genome-wide). After DAWN analysis the FDR *q*=0.0007, which has been corrected for multiple testing and therefore represents much stronger evidence. The additional evidence for association comes from the tight co-expression of *PTEN* with other genes likely involved in risk (Figure 5A). While the complete neurobiological underpinnings of this tightly connected network are not obvious, proteins arising from several of these genes are known to have a common function, neurite extension. For example the protein product of *NCKAP1* plays a role in the protein complex WAVE1, an actin scaffold protein complex that regulates neurite outgrowth [49]. The protein product of *PTEN* likely plays a role in neurite outgrowth by negatively regulating PI3K signaling and affecting neuronal polarization [50]. PTEN could also play a role through the ubiquitin proteasome function [51]. An E3 ubiquitin ligase, Nedd4, and PTEN play complementary roles: Nedd4 knockdown increases levels of PTEN and decreases axon branching; the branching pattern can be recovered by loss of PTEN expression. Cullin RING ligases also play a role in arborization, with loss of the *CUL3* protein product increasing dendritic arborization [51]. Finally, *SMAP1* encodes ARFGAP1, which in part functions to control trafficking of GABA transporter-1, a protein enriched at neurite extensions in certain neurons [52].

*FOXP1* is a transcriptional regulator when it heterodimerizes with FOXP2. Mutations in *FOXP2* have been shown to impair language development, specifically causing developmental apraxia of speech [53,54]. Until recently *FOXP1* was not known to affect language abilities or behavior, but recent reports [7,55,56] suggest disruptions of the gene could cause cognitive dysfunction and ASD, sometimes with language impairment [48]. The evidence, however, is not conclusive. On the basis of the DAWN analyses, *FOXP1* has *q*=0.0083, strong evidence it plays a role in risk, especially when considered with other independent evidence (reviewed in [48]). Notably it is connected directly and with substantial correlation (|*r*|>0.7) to five genes with at least one dnLoF event in ASD probands (Figure 5B), of which two are known ASD risk genes, *SCN2A* and *ANK2*. What role or roles these genes play to effect this coordinated expression is not obvious from the neurobiological literature.

Certain mutations in *SPAST* are known to cause hereditary spastic paraplegia. In some cases, mutations in *SPAST* (also known as *SPG4*) can affect cognitive function and result in developmental delay syndromes [57], as well as incompletely penetrant hereditary spastic paraplegia later in life. Its subnetwork is notable (Figure 5C): *SPAST* is directly connected with 12 genes having at least one dnLoF mutation and three of those genes are known ASD risk genes. The protein product, Spast, severs microtubules and disruption of this function appears to generate a risk for hereditary spastic paraplegia [58]. It also interacts with protrudin to induce axonal neurite outgrowth [59]. This function, together with its direct connections in the network to other genes involved in neurite extension (*NCKAP1* and *CUL3*), suggest at least a portion of this network could affect ASD risk through improper neurite development. *SPAST* and its subnetwork deserve further study for their role in the risk for ASD.

Finally, an interesting case is *VRK1*. Measured by the network score, it is the gene most connected to rASD genes (Additional file 14: Figure S8). VRK1 has diverse functions, arguably most fundamental is regulation of cell cycle. Moreover mutations in *VRK1* have been implicated in neuronal development and maintenance, including cognitive impairment [60]. There is essentially no genetic evidence for its involvement in ASD (TADA *P*=0.572). Therefore, although it is an nASD gene with the highest network score and intriguing neuronal functions, it does not make the rASD list (*q*=0.81).

### Functional interpretation of subnetworks

When looking at the genes comprising the two subnetworks given in Figures 5A,C, it is striking how many genes play some role in the regulation of neurite extension and arborization. Indeed two other predicted risk genes are known to affect this process at a basic level, namely *CDC42EP4* and *CDC42BPB*, both interacting with *CDC42* (Figure 2), which plays a key role in neurite initiation [61,62]. These genes are not highly correlated in the PFC-MSC, so they cannot occur in the same subnetwork, although they are known to serve the same function. *CDC42* activates the *WAVE1* actin scaffold complex, including *NCKAP1* (Figure 5A,C), initiating neurite outgrowth. Notably expression of *CDC42BPB* is highly correlated with expression of *NAV2*, a gene known to impact axonal outgrowth [63]. Being in a subnetwork centered on *NAV2*, its expression is highly correlated with a substantial set of genes (Additional file 5: Table S4), many of which have some role in neurite extension and neuronal arborization, specifically *ATRN*, *CDC42BPB*, *L1CAM*, *MARK4*, *SHANK2*, *MAPT* and *STXBP1*[64-68], although many play other roles in cellular maturation and function.

From this enumeration it appears as if a large fraction of predicted risk genes affect neurite extension and neuronal arborization. On the other hand, it could be that many genes play some role in this critical feature of neuronal development and the number identified here is no larger than we would expect by chance. We therefore formally evaluate the conjecture that the rASD list is enriched with genes related to neurite outgrowth. For this evaluation we turn to unbiased and independent data, specifically functional annotation data from GO. We focus on the GO term for the ‘neuron projection development’ (GO:0031175), which is a biological process and also a synonym for ‘neuron outgrowth’. The list annotated with this term or one of its descendants in the GO hierarchy contains 737 unique genes, including 10 out of 127 rASD genes (*BCL11A*, *FOXP1*, *ITGA5*, *L1CAM*, *MAPT*, *PTEN*, *SPAST*, *STXBP1*, *TBR1* and *TNC*). Compared to random sets of 127 brain-expressed genes, the rASD list is significantly enriched (*P*=0.032, based on 1,000 draws).

**Figure 6:**
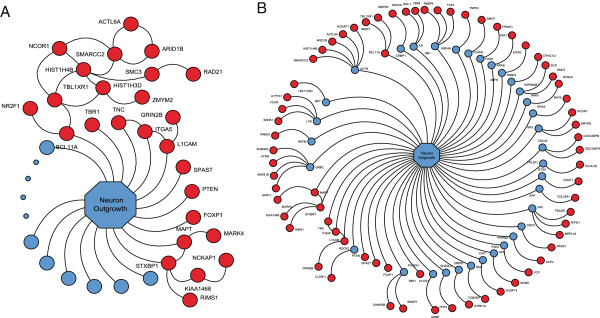
**Predicted risk genes functionally related to neuron outgrowth.(A)** Ten rASD genes are GO-identified for neuron outgrowth and 16 additional genes rASD genes are directly connected via the PPI network. **(B)** Note that 68 rASD genes are either GO-identified for neuron outgrowth or separated in the PPI network from genes annotated by neurite outgrowth by at most one step. PPI, protein-protein interaction; rASD, risk autism spectrum disorder.

Next, it is reasonable to ask if the ten rASD genes GO identified as functionally related to neurite outgrowth are functionally interrelated to other rASD genes. To address this question we identify rASD genes directly connected via the PPI network with the ten GO-identified rASD genes. Using the PPI network provided in [69], we obtained a network of 26 rASD genes (Additional file 15: Table S6), which is significantly enriched (Figure 6A, *P*=0.002, based on 1,000 draws). Finally, when we examine the list of rASD genes separated in the PPI network from genes annotated by neurite outgrowth by at most one step, i.e., rASD genes that interact with a gene annotated by neurite outgrowth, the resulting list includes 68 rASD genes (Additional file 15: Table S6) and is again significantly enriched (Figure 6B, *P*=0.001, based on 1,000 draws).

### DAWN’s limitations

Both genetic evidence and gene expression evidence are required for a risk gene to be identified by DAWN. In the screening stage, a gene can only make it onto the nASD list if it is tightly correlated with multiple genes with moderate genetic evidence for association. Thus a lone gene with a small TADA *P* value cannot make it onto the nASD list. Next in the cleaning stage the subset of the nASD genes that have genetic evidence for association are upgraded to the rASD list. This summary highlights two limitations of DAWN: (i) a risk gene cannot be discovered if there is not some genetic evidence for association and (ii) a risk gene cannot be detected if it does not appear in a network of other risk genes, based on the gene expression network in use. Both of these conditions can fail for a number of reasons, not all of them biologically interesting. For example, the quality of sequencing data could be poor due to low coverage, the power of the genetic test could be poor due to insufficient sample size or the wrong gene expression data could be utilized, yielding an irrelevant network. Other possible limitations to DAWN are biological. For example it is possible that risk for ASD arises from dysfunctional neuronal circuitry that spans distinct regions of the brain, such as from the hindbrain into deep layers of the PFC [70]. Indeed, different genes could contribute to a single circuit and be co-expressed at the circuit level, but not in the same tissue. If this were the case, then coexpression information in a specific tissue for these genes is irrelevant and DAWN would fail to capture this aspect of ASD risk.

For these and other reasons, DAWN cannot possibly capture the bulk of ASD genes. Indeed, as noted in [14], it is unreasonable to predict that the mid-fetal PFC-MSC is the only nexus for ASD risk genes. Underscoring this observation, strikingly absent from the rASD set are some genes known to affect risk for ASD. For instance the neurexins (*NRXN1*, *NRXN2* and *NRXN3*) and neuroligins (*NLGN1*, *NLGN3* and *NLGN4X*) are either known to affect risk or have been implicated in risk on the basis of rare sequence mutations and copy number variants (reviewed in [71]). These proteins pair across the synapse to play a critical role in its development [72]. There could be several reasons why these genes are not found in the rASD list. They might not be captured effectively by current exome sequencing methods, in which case the genetic data cannot produce small *P* values. Indeed TADA *P* values for all of these genes are unimpressive [12]. If many genes underlie risk for ASD, as recent analyses suggest, the power to detect a ‘genetic signal’ for association is low for each particular gene without very large samples. In addition, DAWN draws strength from the connectedness of genes on the basis of their co-expression. If genes do not have a substantial network score, then they will not be included in the rASD list. In this regard, only *NLGN4X* and *NLGN1* rank highly for their network scores. Notably the inter-correlations amongst neuroligin and neurexin gene expression are not large in these data (Additional file 16: Table S7), suggesting they have different roles in the development of the mid-fetal PFC-MSC.

We believe it is impossible to identify all risk genes in one analysis, regardless of the methods employed. To enhance power, one could parse the literature for evidence of a gene’s impact on risk and somehow include that evidence in the DAWN analysis. The downside to that approach is that it will be difficult and somewhat subjective to score the evidence from studies that have different experimental designs and results. Specifically, the way the data are collected could affect the estimate of risk parameters for specific genes; for example, syndromic genes, which affect multiple systems, could be over-represented in the literature, potentially exaggerating their importance for ASD risk.

### Extensions to DAWN

On the other hand, there is unbiased information that is not yet built into the algorithm, such as chromatin modification, other features of gene regulation and other sources of information regarding association with disease. We are working on extensions of DAWN to accommodate these kinds of data. There is also potentially biased but valuable information that should be evaluated for modeling. For example, we noted that a significant number of risk genes are involved in the regulation of neurite extension or arborization. While it would be challenging, this kind of information would ideally be incorporated into an algorithm such as DAWN.

An additional concern for any meta-analytic approach like DAWN is data quality, both from gene expression and genome sequencing. Poor quality assessment of gene expression obscures the construction of gene networks. In addition, unless whole-exome or whole-genome sequencing becomes very cost effective and highly representative of genomic content, there will always be genes with poor coverage from methods that target the entire exome or genome. We have assumed the missing information is random, with respect to risk for ASD, but it still reduces the ability to predict risk genes. Interestingly alternative methods that capture those missing exons would benefit from analyses such as DAWN. The results from DAWN, specifically the nASD genes and their network scores, when combined with information about coverage of the sequencing experiments, provides key information about which genes should be targeted with alternative sequencing methods (e.g., [17]) because they are closely integrated with genes that affect risk.

In the very near future, studies will refine the data relevant to gene expression and to the genetics of ASD. The genetic data are expected to change dramatically during the next few years [1]. Thus, we expect the rASD gene set and its associated subnetworks to be refined and expanded with these new data. To speed the gene discovery process, candidate gene validation studies can be applied to large samples of trios using the results from DAWN to guide in gene selection.

### Synopsis of results

Using the DAWN algorithm to integrate gene co-expression and genetic data, we identified over a hundred genes with compelling evidence they affect risk for ASD (Table 1). Our analyses also identified subnetworks of genes likely to be involved in risk for ASD, and for the majority of genes included there is no strong evidence for risk from genetic results alone (Figures 2 and 4). Our analyses build directly on the results from [14] because we target the mid-fetal PFC-MSC, where a striking and highly significant co-expression of genes is implicated in ASD risk on the basis of *de novo* mutations. It is reasonable to predict that such strong prior information is essential for the success of methods such as DAWN. Similarly, refined co-expression networks obtained from larger samples and from RNAseq experiments should improve the performance of DAWN, while expression data from additional brain regions could yield additional findings [14].

The results from DAWN also clarify the neurobiology of ASD. A prominent theory for its etiology proposes it is caused by aberrant connectivity of neuronal circuits due to intrinsically abnormal synapses [73,74]. Indeed, the sheer number of ASD genes playing a key role in synaptic development or function strongly support this theory. In this regard, the subnetworks around *PTEN* (Figure 5A), *SPAST* (Figure 5C) and *NAV2*(Additional file 5: Table S4) are quite intriguing. Portions of these networks play key roles in neuritic outgrowth, arborization, guidance and terminal specification of both axons and dendrites. Moreover, when we evaluate the entire list of genes implicated in risk (Table 1), we find highly significant enrichment of these genes for involvement in neuron projection development. Recent support for enrichment also comes from a bioinformatics analysis of common variants potentially affecting ASD risk [75]. Proper circuits can only be achieved when synapses function properly and when they exist in the appropriate numbers, distributions and specificities [76]. In other words, the wiring diagram is as important to neural circuit function as the quality of its connections. Thus a hypothesis to explain these subnetworks is that they converge on mediation of coordinated neurite development and that risk for ASD arises from disorganized patterns of arborization in addition to the often-described synaptic dysfunction [77]. This hypothesis is consistent with a common feature of subjects with ASD, namely a slightly but stochastically larger brain than expected [78], consistent with overgrowth and/or abnormal synaptic pruning.

## Conclusions

DAWN offers a general approach to gene discovery, which can be applied to many complex disorders. The algorithm leverages genetic and gene expression data effectively to predict probable risk genes and subnetworks. Validation studies demonstrate that DAWN is successful in predicting the genes that will accumulate new dnLoF mutations better than any existing methods, underscoring the high likelihood that DAWN is finding true risk genes for ASD. The set of risk genes reported here provides further support for the theory that neurite extension and neuronal arborization play a key role in risk for ASD. Currently DAWN’s findings are limited by the power of test statistics derived from available samples with exome sequencing. And yet this algorithm has already yielded a rich harvest of potential risk genes. Emerging ASD sequencing data, based on larger sample sizes, will greatly improve the quality of genetic information going into the algorithm, which will further enhance the power of DAWN to identify subnetworks of risk genes.

## Abbreviations

ASD: autism spectrum disorder; BP: biological process; DAWN: detecting association with networks; dnLoF: *de novo* loss of function; FDR: false discovery rate; FET: Fisher’s exact test; GO: gene ontology; LoF: loss of function; MF: molecular function; MGI: mouse genome information; MIPS: molecular inversion probe sequencing; MP: molecular phenotype; nASD: network autism spectrum disorder; PFC-MSC: prefrontal and motor-somatosensory neocortex; PPI: protein-protein interaction; rASD: risk autism spectrum disorder; TADA: transmission and *de novo* association; WGCNA: weighted correlation network analysis.

## Competing interests

The authors declare that they have no competing interests.

## Authors’ contributions

LL conceived of the analysis, developed and refined the DAWN statistical software, implemented the analysis, wrote the manuscript and produced graphics. JL conceived of the analysis and commented on the manuscript. SJS and AJW implemented the analysis, produced graphics, and revised and edited the manuscript. YK and AEC implemented the analysis and produced graphics. LK, CL and XH implemented the analysis. ML compiled and analyzed the data. RAM and AM revised and edited the manuscript. JPN, NS and MWS conceived the study, provided financial support and revised and edited the manuscript. KM and JB conceived the study and wrote the manuscript. BD and KR conceived of the analysis, provided financial support and wrote the manuscript. All authors read and approved the final manuscript.

## Supplementary Material

Additional file 1 **Table S1.** Summary of all available gene expression samples from [16]. Click here for file

Additional file 2 **Figure S1.** Network analysis of gene expression in the frontal cortex (PFC-MSC) and distribution of risk ASD (rASD) genes within modules for periods 3–5 and 4–6. **(A)** Dendrogram produced by hierarchical clustering of gene co-expression in periods 4–6 using WGCNA. Modules of co-expressed genes are delineated by color. In the second depiction of the dendrogram, rASD genes are highlighted with a color according to module membership; other genes are colored in gray. Counts are number of rASD genes per module. Most rASD genes fall in tight clusters within modules, and yet they fall in many distinct modules.**(B)** As above, but for periods 3–5. Click here for file

Additional file 3 **Table S2.** Sets of overlapping gene modules formulated using four criteria: correlation at developmental periods 3–5 and 4–6, both with modules created using powers 1 and 6 to define the adjacency matrix in WGCNA. By varying the definition of adjacency slightly we capture more of the features of the gene clusters. *Median (1st, 3rd quantile). Click here for file

Additional file 4 **Table S3.** Number of modules in periods 3–5 and periods 4–6 analysis. Ideally modules successfully split the genes into clustered subsets with strong correlations within a module and weak correlations across modules. Not surprisingly this is an imperfect process and modules create some artificial boundaries that separate genes with fairly strong levels of correlation. One method for module construction involves choosing a power that produces a scale-free topology; however, this choice yielded a large number of small modules that was unsuitable for the planned analysis. We chose powers 1 and 6 to span a range of plausible modules. Power 6 yielded smaller and more numerous modules than power 1 for both time periods; moreover, many of the power 6 modules were quite small and not suitable for the planned network analysis (Additional file 2: Figure S1). For power 6, many of these small modules could be successfully merged together based on the eigengenes. In contrast, power 1 produced larger modules and merging via eigengenes led to one very large module that was also not suitable for the network analysis (Additional file 2: Figure S1). To obtain a reasonable collection of mid-sized modules we use the unmerged modules for power 1 and the merged modules for power 6. Notably, this choice produced a similar number of modules for each choice of power and many of these modules were of similar size. Click here for file

Additional file 5 **Table S4.** Statistics for all genes analyzed in periods 3–5 and 4–6. The summary tab is a summary of results over all four modular representations (powers 1 and 6 for periods 3–5 and 4–6). For the rASD and nASD columns, a gene was labeled ‘yes’ if it was identified in any of the four module sets. min_FDR (network score) is the minimum (maximum) value over all four module sets. In the annotation column, 0, 1 or 2 represents a gene with 0, 1, or at least 2 identified dnLoF mutations, respectively. Tabs period4–6 and period3–5 provide similar information for each separate time period. The rASD_p4-6 and rASD_p3-5 tabs provide validation scores for rASD genes identified in the analysis of periods 4–6 and 3–5, respectively. The rASD_correlation tab gives the set of neighboring genes for all rASD genes (i.e., rASD genes with |*r*|>.7). Click here for file

Additional file 6 **Figure S2.** Identifying ASD genes and subnetworks by a network analysis of gene expression and association statistics. (A) Gene co-expression networks derive from pairwise correlations of gene expression. After sorting genes into modules by using WGCNA, some genes cluster into highly connected units, called supernodes, which are identified by cutting the hierarchical tree at.75. (B) Each node is represented by a Z-score derived from the TADA *P* value. Supernodes are represented by the score associated with the minimum *P* value of all genes in the node. An adjacency matrix connects nodes with absolute correlation greater than.7. (C) A hidden Markov random field model is used to model correlation of the *Z*-scores across the gene network. (D) The modeling process yields subnetworks with evidence for involvement in risk for ASD, and the entire set of genes involved in associated subnetworks are called network ASD genes (nASD). On the left, red balls indicate nodes with relatively large *Z*-scores, prior to network analysis. On the right, red balls delineate nodes that are identified as nASD genes based on clustering of signal. Unconnected nodes tend to turn blue and tightly connected nodes turn red. The top module displays a tightly clustered signal; the bottom one is unclustered, and no nASD genes are identified. (E) A small module illustrates details. (F) To identify genes likely to affect risk for ASD (rASD), all nASD genes are examined further based on their *Z*-scores. (G) For large supernodes, risk genes are determined based on clustering of signal in the *Z*-score within a supernode; for small supernodes and singleton nodes the delineation is determined purely by *Z*-score. Click here for file

Additional file 7 **Text S1.** Detailed description of the DAWN algorithm. Click here for file

Additional file 8 **Figure S3.** Distribution of network scores across genes from the frontal cortex. **(A)** Box plots of network scores for genes divided into three categories: non-nASD genes, nASD genes (excluding rASD genes) and rASD genes. Results are displayed for periods 4–6 (yellow) and 3–5 (orange). **(B)** Correlation of network scores by time period for the set of nASD gene found in both time periods. The red dashed line is the diagonal line *y*=*x*. Click here for file

Additional file 9 **Figure S4.** Distribution of *Z*-scores based on TADA *P* values for all nASD genes. Genes that are also rASD genes are colored in red, and the remainder are colored dark cyan. Click here for file

Additional file 10 **Figure S5.** Discovery rate of genes with *de novo* LoF mutations as the signal becomes more diluted. Two dilution experiments were performed: **(A)** weakening the *P* value signal and **(B)** weakening the correlation structure. The number of *de novo* genes identified (*#*dnLoF) is plotted in blue, as a function of the dilution of the signal, ranging from 0 to 100%, and the HMRF parameter *c*, which measures the strength of clustering of signal in the networks, is plotted in orange. The standard error of the estimates is indicated with error bars. Click here for file

Additional file 11 **Table S5.** Summary of *de novo* variants identified for 44 selected genes for the MIPS experiment. Click here for file

Additional file 12 **Figure S6.** PPI network of all rASD genes. The edge information was obtained using DAPPLE [79]. Click here for file

Additional file 13 **Figure S7.** Enrichment analysis using genes from the clusters shown in Figure 4 with the ChEA, Wikipathways, GO_biological Process, MGI_Mouse Phenotype and Human Gene Atlas gene-set libraries. Click here for file

Additional file 14 **Figure S8.** Subnetwork of rASD genes for *VRK1*. This gene has the highest network score among all nASD genes, but this gene, which has no signal of association in its TADA score, was not identified as an rASD gene. Click here for file

Additional file 15 **Table S6.** The list of GO-identified rASD genes. Click here for file

Additional file 16 **Table S7.** Correlations amongst neurexin and neuroligin genes for periods 4–6 (top) and 3–5 (bottom). Click here for file
