# Supplementary figures and images for: DAWN: a framework to identify autism genes and subnetworks using gene expression and genetics
Source: Mol Autism. 2014 Mar 6;5:22. doi: 10.1186/2040-2392-5-22 (PMC4016412; doi:10.1186/2040-2392-5-22)

A

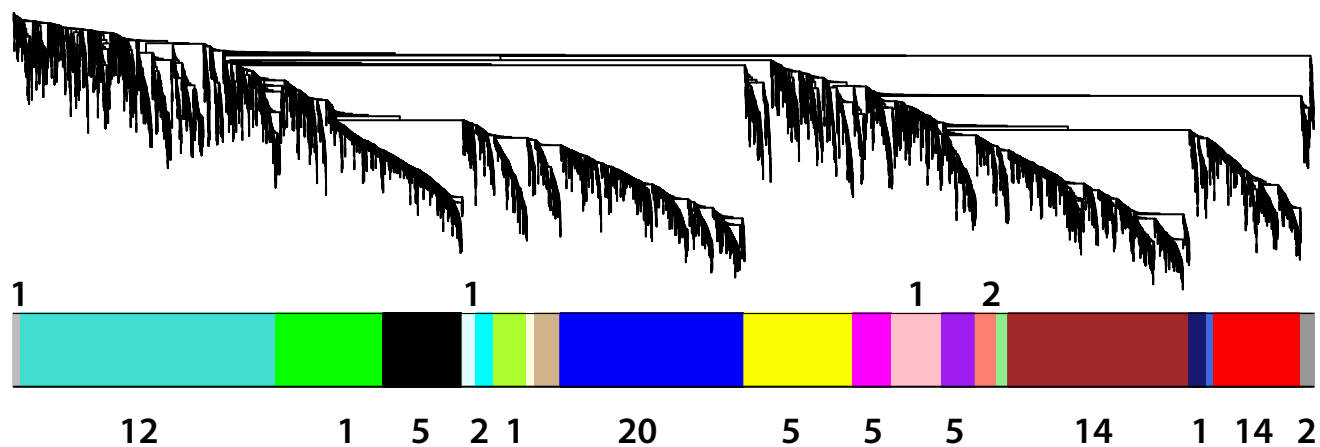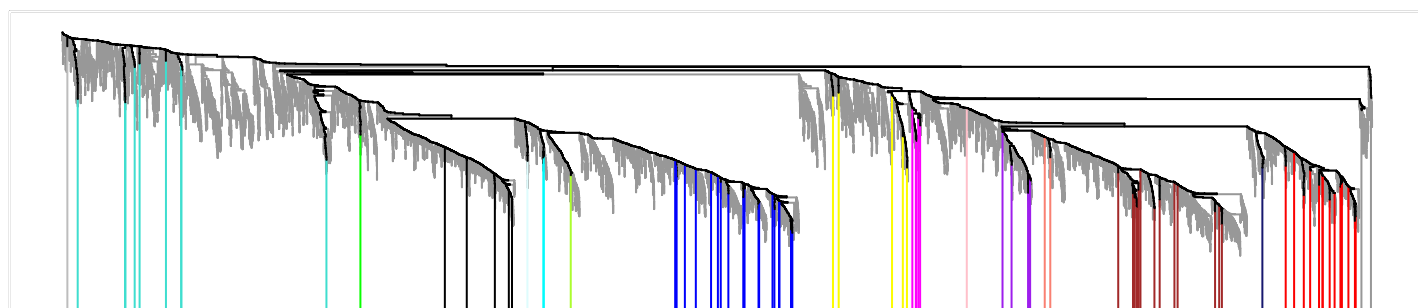

B

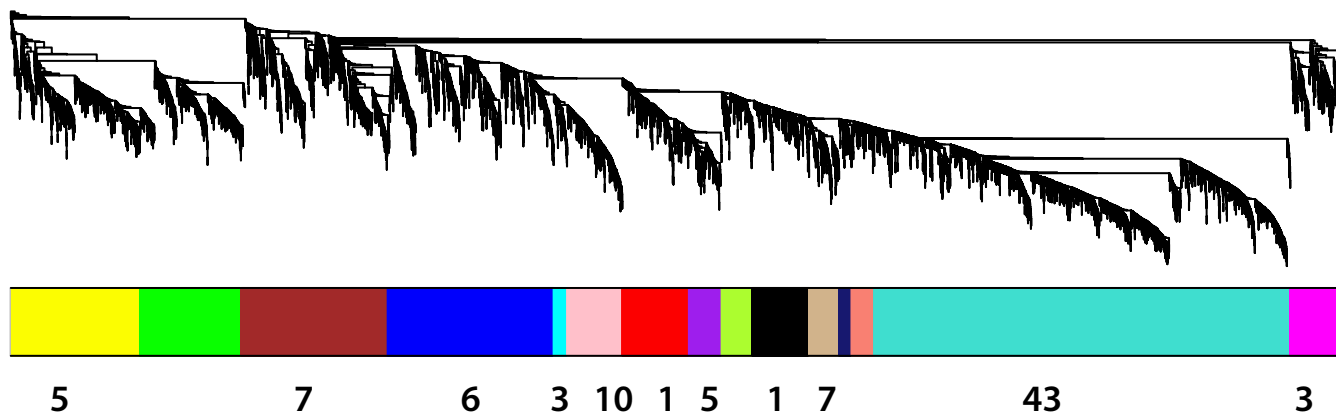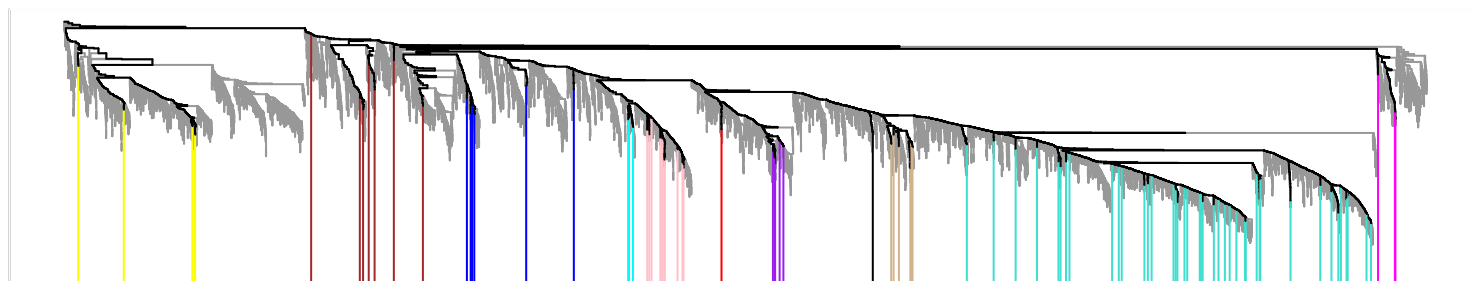

Supplement: Additional file 2 — Figure S1. Network analysis of gene expression in the frontal cortex (PFC-MSC) and distribution of risk ASD (rASD) genes within modules for periods 3–5 and 4–6. (A) Dendrogram produced by hierarchical clustering of gene co-expression in periods 4–6 using WGCNA. Modules of co-expressed genes are delineated by color. In the second depiction of the dendrogram, rASD genes are highlighted with a color according to module membership; other genes are colored in gray. Counts are number of rASD genes per module. Most rASD genes fall in tight clusters within modules, and yet they fall in many distinct modules.(B) As above, but for periods 3–5. [file 2040-2392-5-22-S2.pdf]

# Algorithm for identifying ASD genes and networks

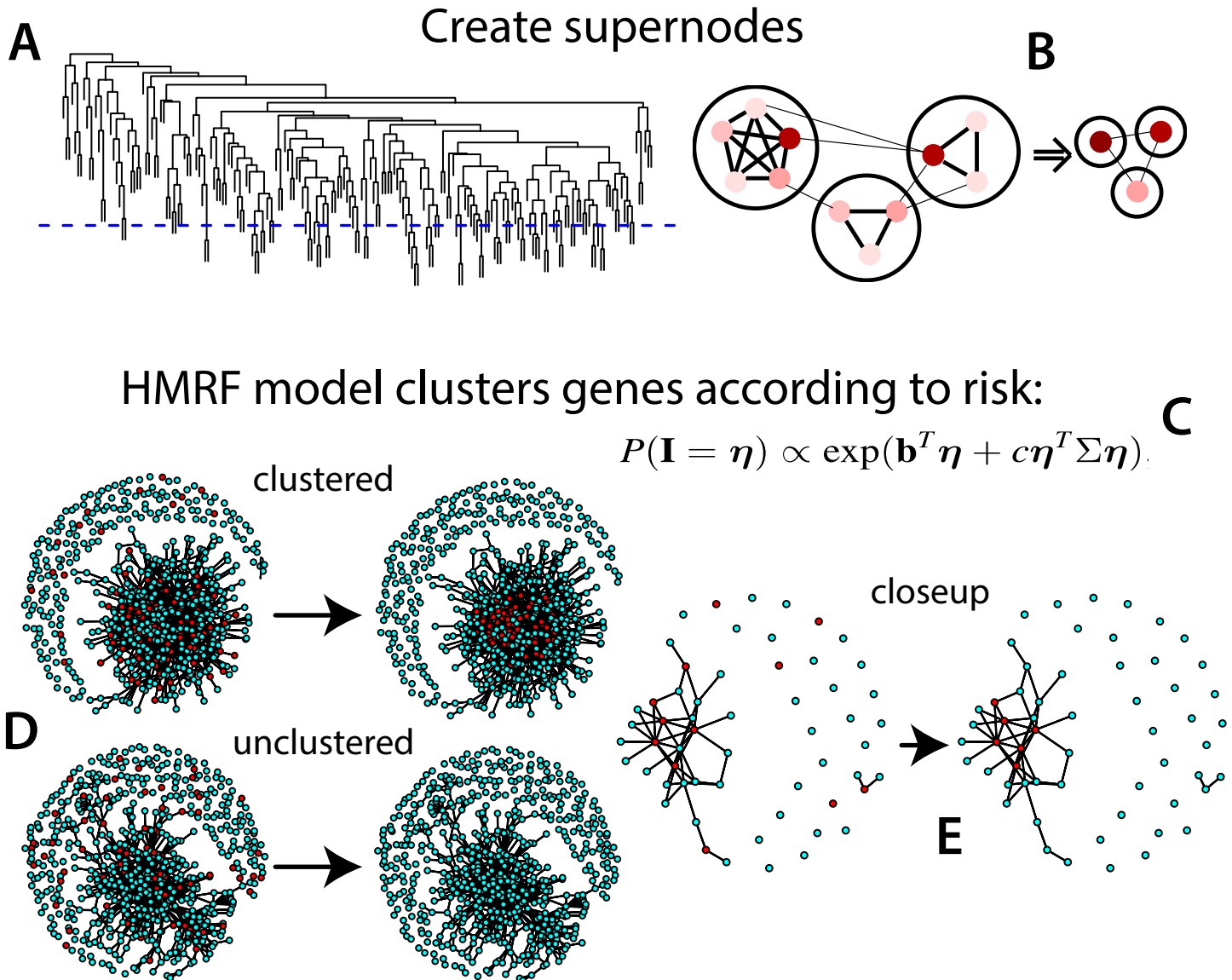

Network genes (nASD) -> probable risk genes (rASD)

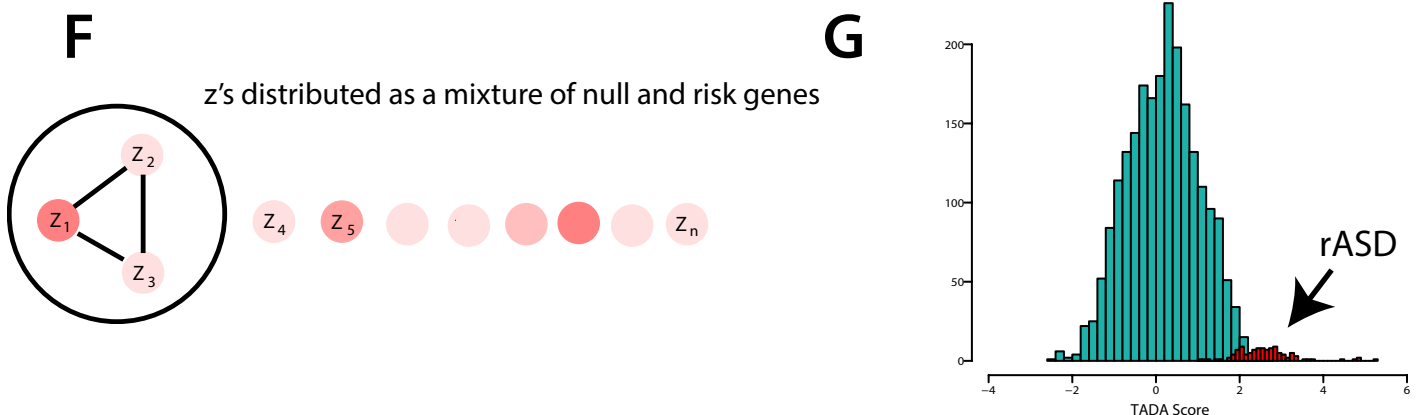

Supplement: Additional file 6 — Figure S2. Identifying ASD genes and subnetworks by a network analysis of gene expression and association statistics. (A) Gene co-expression networks derive from pairwise correlations of gene expression. After sorting genes into modules by using WGCNA, some genes cluster into highly connected units, called supernodes, which are identified by cutting the hierarchical tree at.75. (B) Each node is represented by a Z-score derived from the TADA P value. Supernodes are represented by the score associated with the minimum P value of all genes in the node. An adjacency matrix connects nodes with absolute correlation greater than.7. (C) A hidden Markov random field model is used to model correlation of the Z-scores across the gene network. (D) The modeling process yields subnetworks with evidence for involvement in risk for ASD, and the entire set of genes involved in associated subnetworks are called network ASD genes (nASD). On the left, red balls indicate nodes with relatively large Z-scores, prior to network analysis. On the right, red balls delineate nodes that are identified as nASD genes based on clustering of signal. Unconnected nodes tend to turn blue and tightly connected nodes turn red. The top module displays a tightly clustered signal; the bottom one is unclustered, and no nASD genes are identified. (E) A small module illustrates details. (F) To identify genes likely to affect risk for ASD (rASD), all nASD genes are examined further based on their Z-scores. (G) For large supernodes, risk genes are determined based on clustering of signal in the Z-score within a supernode; for small supernodes and singleton nodes the delineation is determined purely by Z-score. [file 2040-2392-5-22-S6.pdf]

A

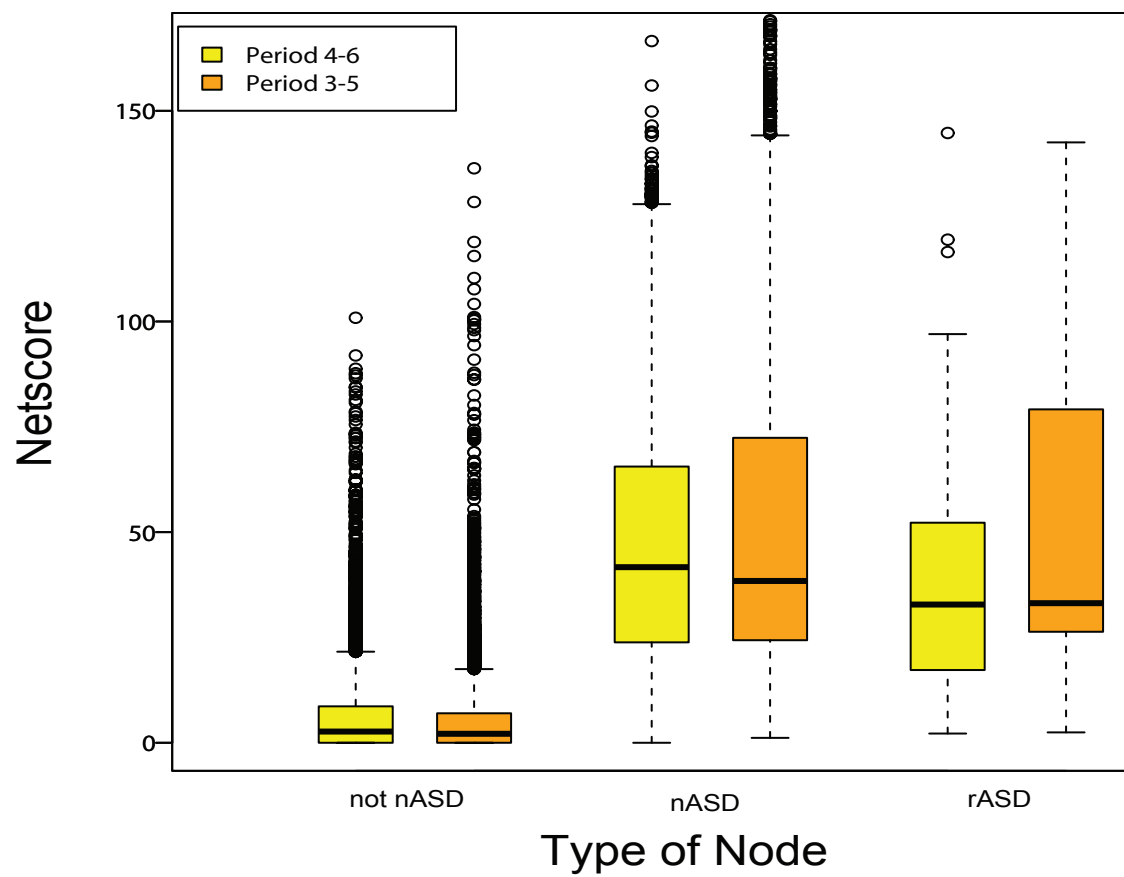

B

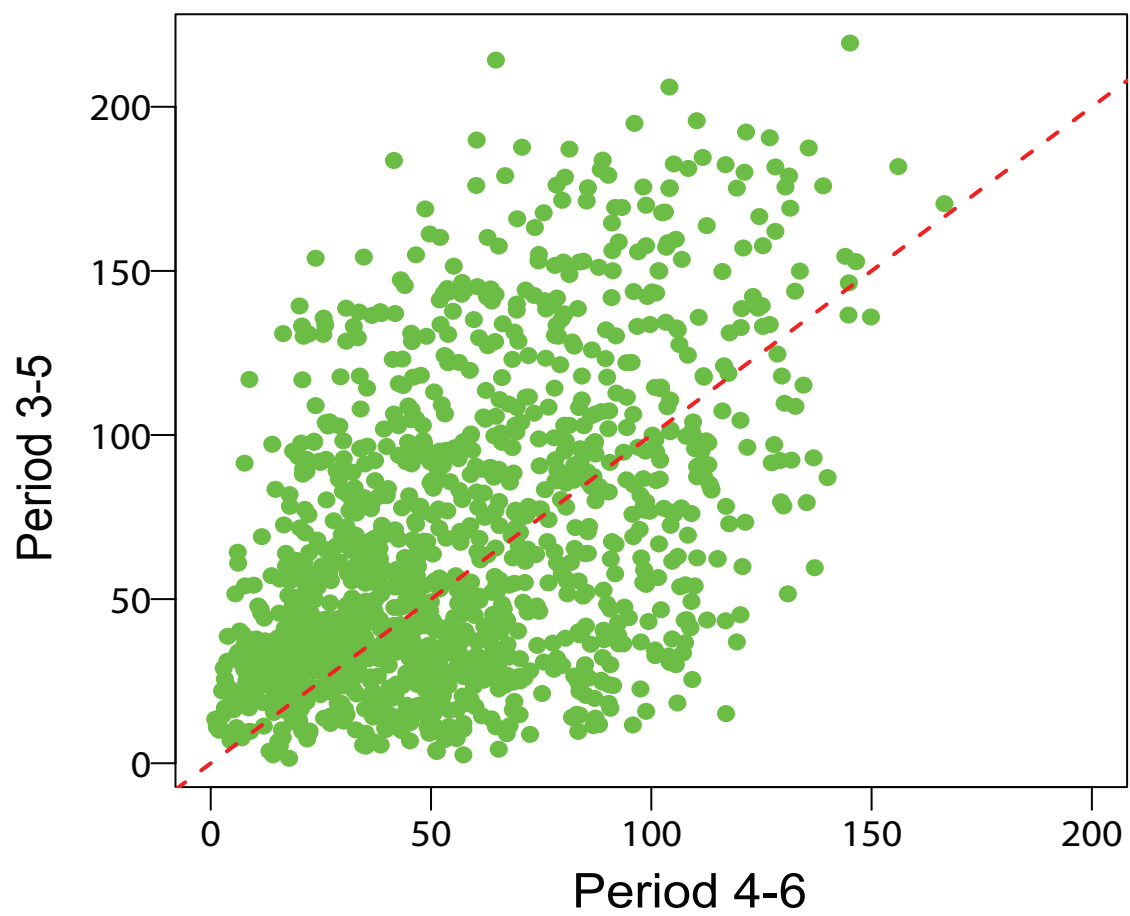

Supplement: Additional file 8 — Figure S3. Distribution of network scores across genes from the frontal cortex. (A) Box plots of network scores for genes divided into three categories: non-nASD genes, nASD genes (excluding rASD genes) and rASD genes. Results are displayed for periods 4–6 (yellow) and 3–5 (orange). (B) Correlation of network scores by time period for the set of nASD gene found in both time periods. The red dashed line is the diagonal line y=x. [file 2040-2392-5-22-S8.pdf]

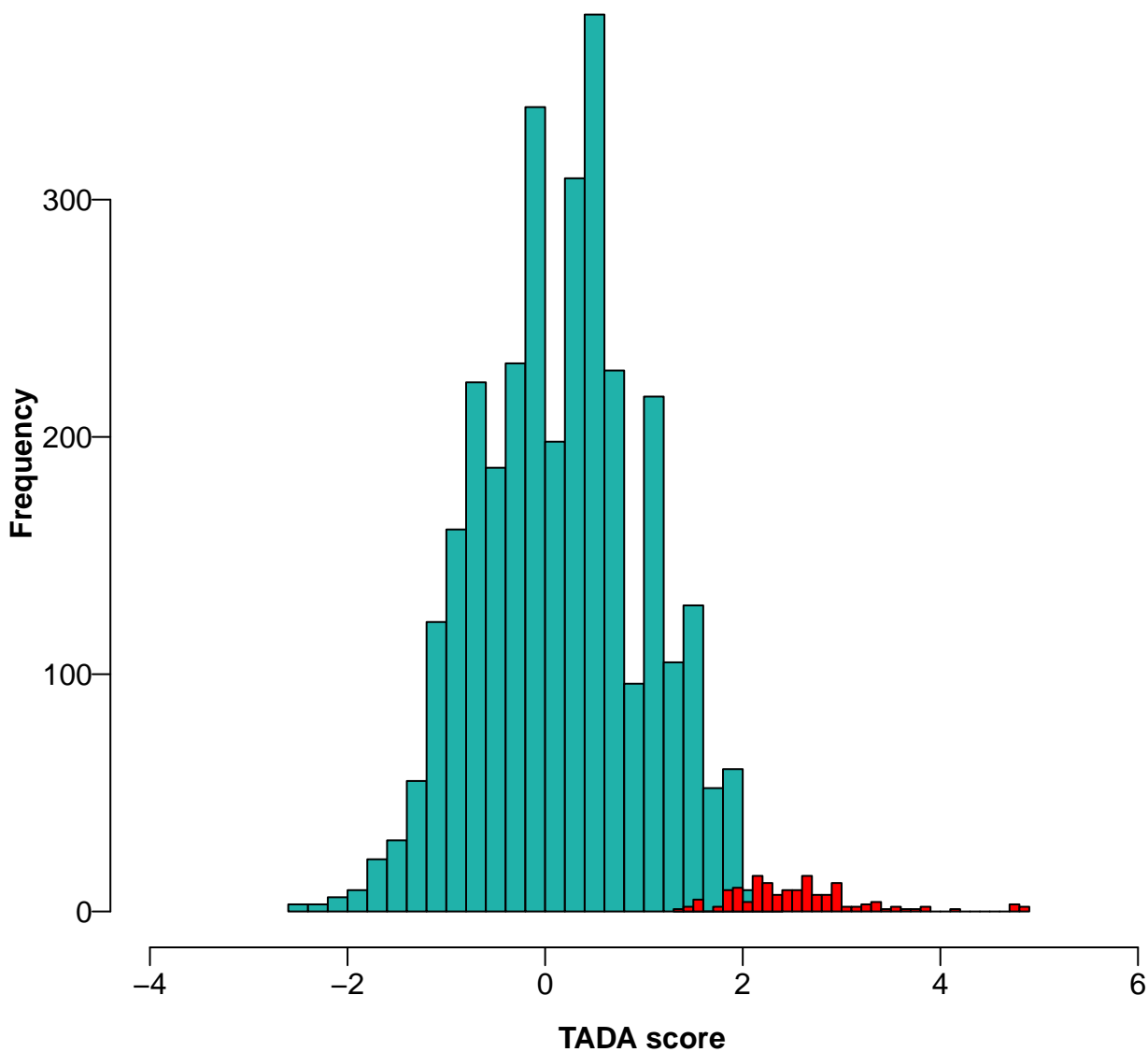

Supplement: Additional file 9 — Figure S4. Distribution of Z-scores based on TADA P values for all nASD genes. Genes that are also rASD genes are colored in red, and the remainder are colored dark cyan. [file 2040-2392-5-22-S9.pdf]

**A**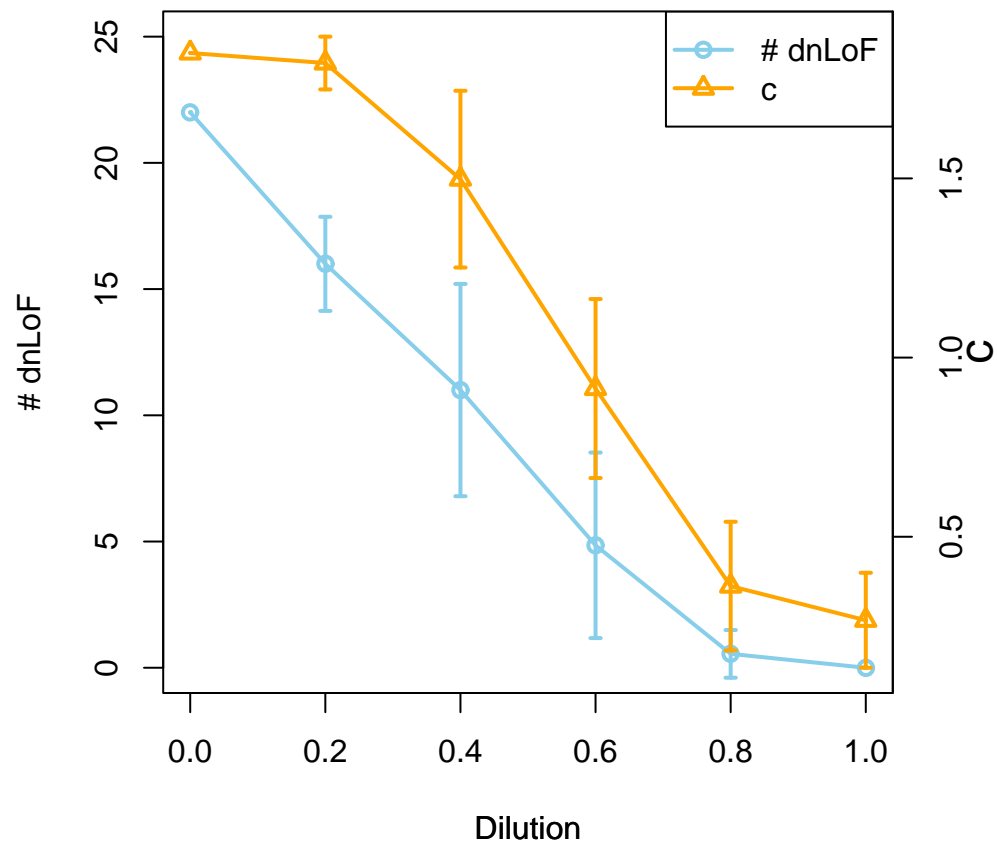**B**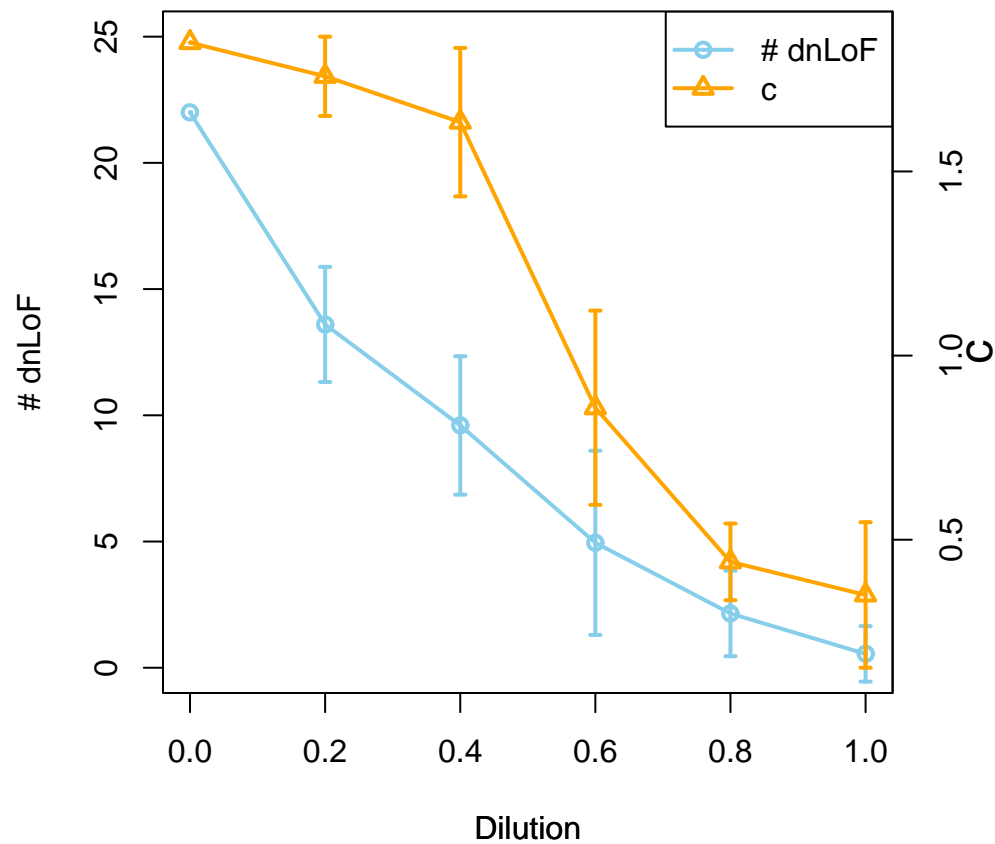

Supplement: Additional file 10 — Figure S5. Discovery rate of genes with de novo LoF mutations as the signal becomes more diluted. Two dilution experiments were performed: (A) weakening the P value signal and (B) weakening the correlation structure. The number of de novo genes identified (#dnLoF) is plotted in blue, as a function of the dilution of the signal, ranging from 0 to 100%, and the HMRF parameter c, which measures the strength of clustering of signal in the networks, is plotted in orange. The standard error of the estimates is indicated with error bars. [file 2040-2392-5-22-S10.pdf]

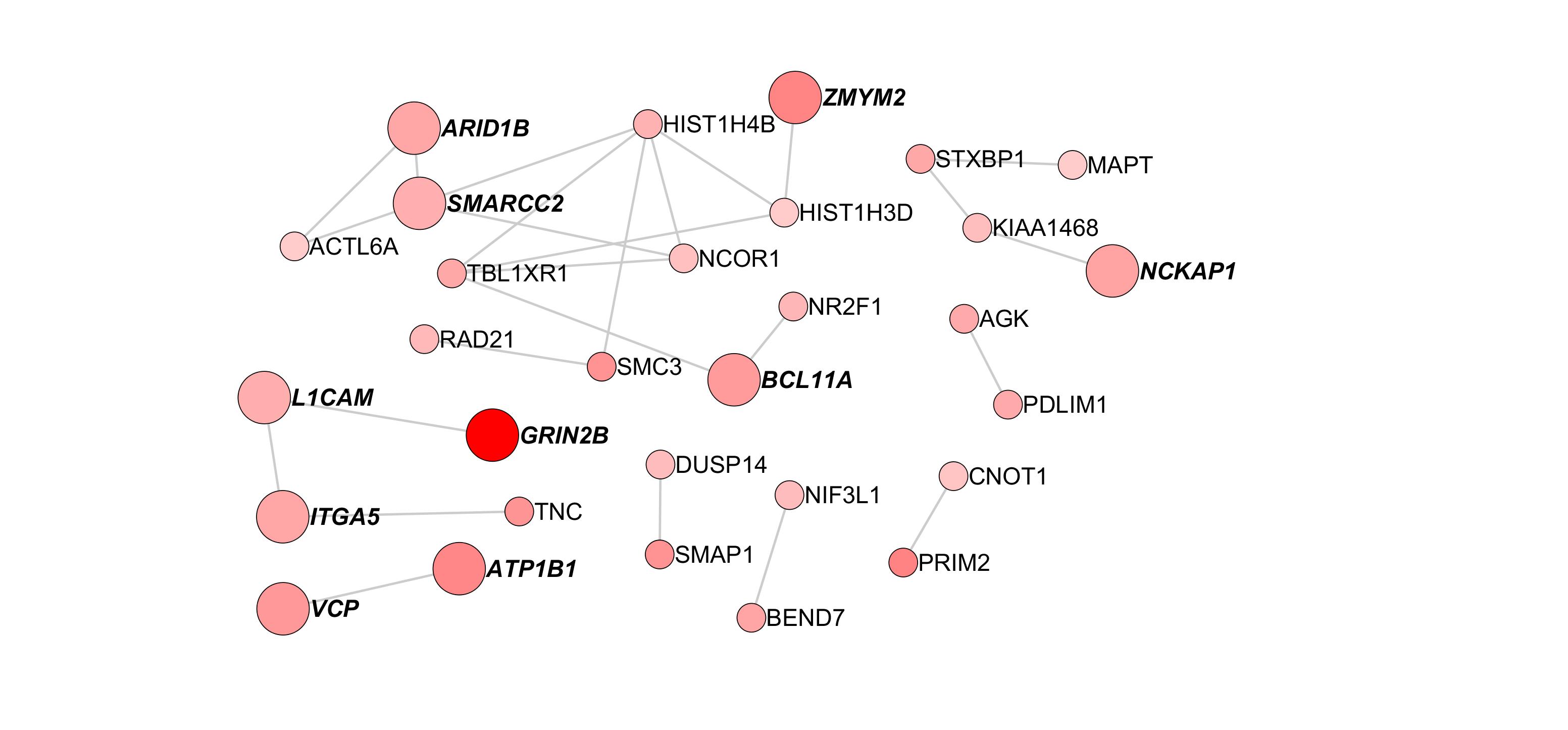

Supplement: Additional file 12 — Figure S6. PPI network of all rASD genes. The edge information was obtained using DAPPLE [79]. [file 2040-2392-5-22-S12.jpeg]

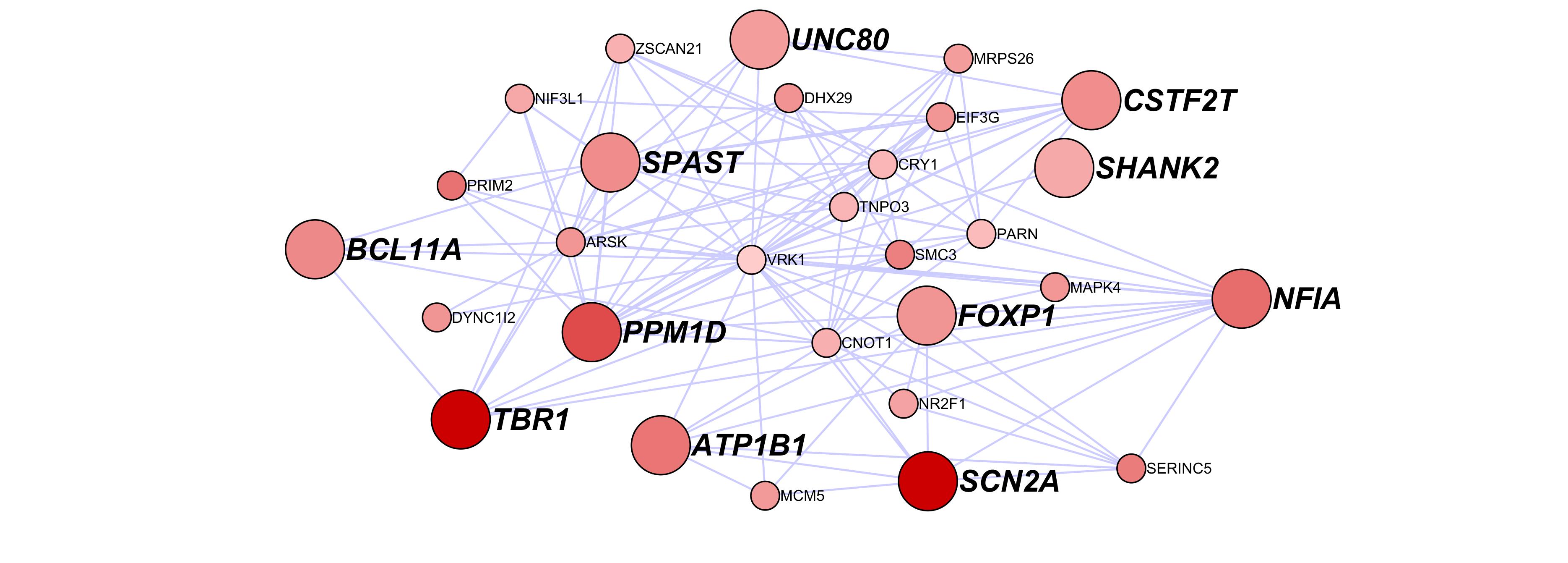

Supplement: Additional file 14 — Figure S8. Subnetwork of rASD genes for VRK1. This gene has the highest network score among all nASD genes, but this gene, which has no signal of association in its TADA score, was not identified as an rASD gene. [file 2040-2392-5-22-S14.jpeg]
