# Supplementary material for: DAWN: a framework to identify autism genes and subnetworks using gene expression and genetics
Source: Mol Autism. 2014 Mar 6;5:22. doi: 10.1186/2040-2392-5-22 (PMC4016412; doi:10.1186/2040-2392-5-22)
Supplement: Additional file 3 — Table S2. Sets of overlapping gene modules formulated using four criteria: correlation at developmental periods 3–5 and 4–6, both with modules created using powers 1 and 6 to define the adjacency matrix in WGCNA. By varying the definition of adjacency slightly we capture more of the features of the gene clusters. *Median (1st, 3rd quantile). [file 2040-2392-5-22-S3.docx]

|  | Period 3-5 | | Period 4-6 | |
| --- | --- | --- | --- | --- |
|  | Power1 | Power6 | Power1 | Power6 |
| No. modules | 21 | 20 | 16 | 15 |
| No. genes in modules | 9450 | 9450 | 9348 | 9348 |
| No. disconnected genes | 5201 | 5201 | 5303 | 5303 |
| Largest module | 2000 | 1770 | 2668 | 1991 |
| Median genes per module | 229 | 253 | 358 | 438 |
| Smallest module | 50 | 39 | 82 | 37 |
| No. supernodes | 1061 | 1117 | 1042 | 1100 |
| No. single gene nodes | 4475 | 4277 | 4593 | 4372 |
| Median no. genes per node | 4.2 | 4.1 | 4.0 | 4.0 |
| No. nASD genes | 2333 | | 2323 | |
| nASD netscores | 41 (24, 65)^*^ | | 38 (24, 72) ^*^ | |
| Non-nASD netscores | 2 (0,9) ^*^ | | 2 (0,7) ^*^ | |
| No. rASD (before validation) | 110 | | 104 | |
| No. rASD (after validation) | 92 | | 92 | |
