# Supplementary material for: DAWN: a framework to identify autism genes and subnetworks using gene expression and genetics
Source: Mol Autism. 2014 Mar 6;5:22. doi: 10.1186/2040-2392-5-22 (PMC4016412; doi:10.1186/2040-2392-5-22)
Supplement: Additional file 4 — Table S3. Number of modules in periods 3–5 and periods 4–6 analysis. Ideally modules successfully split the genes into clustered subsets with strong correlations within a module and weak correlations across modules. Not surprisingly this is an imperfect process and modules create some artificial boundaries that separate genes with fairly strong levels of correlation. One method for module construction involves choosing a power that produces a scale-free topology; however, this choice yielded a large number of small modules that was unsuitable for the planned analysis. We chose powers 1 and 6 to span a range of plausible modules. Power 6 yielded smaller and more numerous modules than power 1 for both time periods; moreover, many of the power 6 modules were quite small and not suitable for the planned network analysis (Additional file 2: Figure S1). For power 6, many of these small modules could be successfully merged together based on the eigengenes. In contrast, power 1 produced larger modules and merging via eigengenes led to one very large module that was also not suitable for the network analysis (Additional file 2: Figure S1). To obtain a reasonable collection of mid-sized modules we use the unmerged modules for power 1 and the merged modules for power 6. Notably, this choice produced a similar number of modules for each choice of power and many of these modules were of similar size. [file 2040-2392-5-22-S4.docx]

Table S3. Number of modules in period 3-5 and period 4-6 analysis

|  | Period 3-5 | | Period 4-6 | |
| --- | --- | --- | --- | --- |
|  | Power1 | Power6 | Power1 | Power6 |
| Merged | 13 | 20 | 8 | 15 |
| Unmerged | 21 | 40 | 16 | 26 |
