# Supplementary material for: DAWN: a framework to identify autism genes and subnetworks using gene expression and genetics
Source: Mol Autism. 2014 Mar 6;5:22. doi: 10.1186/2040-2392-5-22 (PMC4016412; doi:10.1186/2040-2392-5-22)
Supplement: Additional file 7 — Text S1. Detailed description of the DAWN algorithm. [file 2040-2392-5-22-S7.pdf]

Text S1: Detecting Association With Networks (DAWN) Analysis

We assume that n nodes (genes) are under investigation and each node is either risk related ($I = 1$) or not ($I = 0$). The goal is to learn the status of $\mathbf{I} = (I_1, ..., I_n)$. For each node, a TADA test statistic P value is available based entirely on genetic information. Without using any additional information about gene networks, He et al. (2013) used the false discovery rate (FDR) (Benjamini & Hochberg 1995) to determine which genes were risk genes. Our aim is to maintain control of FDR and yet improve the power of this test. It is apparent from the literature that ASD genes tend to be clustered with respect to correlated gene expression and regulatory networks. We use this prior information about the correlation among hidden variables $\mathbf{I}$ in our model to gain power.

The TADA p-values can be converted to Z-scores, $\mathbf{Z} = (Z_1, ..., Z_n)$, to obtain a measure of the evidence of ASD association for each gene. It follows immediately that each of the Z-scores under the null hypothesis ($I = 0$) has a standard normal distribution. We assume that under the alternative ($I = 1$) the Z-scores approximately follow a shifted normal distribution, with a mean μ and variance σ^2. Further we assume that the Z-scores are conditionally independent given the hidden indicators $\mathbf{I}$.

We model the clustering of association signals with respect to gene expression networks using a hidden Markov random field (HMRF) model. Based on the co-expression network we have an adjacency matrix Σ. We assume that the distribution of $\mathbf{I}$ follows an Ising model. The Ising model implies the following conditional probability model for the nodes in the network: the conditional probability the j'th node is a risk node, given the status of the adjacent nodes, is

$$P(I_j = 1 | I_{N_j}) \propto \exp\left(b + c \sum_{i \in N_j} I_i I_j\right),$$

where N_j is the neighborhood of node j based on the adjacency matrix Σ, b models the overall fraction of ASD risk nodes, $c > 0$ reflects the enhanced connectivity of risk status with respect to co-expression of genes. If c is near 0, it suggests that ASD risk genes are not clustered with respect to co-expression of genes.

Each step of DAWN algorithm is summarized by the following:

DAWN Algorithm

Input: an $n \times n$ matrix of pairwise correlation of gene expression for n genes, and the TADA P value of each gene.

Output: a risk ASD (rASD) gene list, posterior probabilities and FDR values of each gene.

1. Cluster genes into modules by applying WGCNA (Langfelder and Horvath, 2008) to the input pairwise correlation matrix.

2. Within each module, group tightly correlated genes to supernodes (absolute correlation larger than .75).

3. Within each module, an adjacency matrix Σ connects nodes (and supernodes) with high absolute correlation (average linkage smaller than .3).

4. Assign Z-score derived from the TADA P value to each node. Supernodes are represented by the score associated with the minimum P value of all genes contained in the node.

5. A HMRF model is applied to model correlation of the Z-scores across the gene network in each module. Nodes which have estimated hidden state $\widehat{I} = 1$ are selected as network ASD (nASD) nodes. See Section 1 for the details about the parameters estimation of HMRF model.

6. All nASD genes are examined further by a hierarchical model based on their Z-scores to identify genes likely to affect risk for ASD (rASD genes). Genes with FDR less than 0.05 are selected into the rASD gene list. See Section 2 for details about the hierarchical model and parameter estimation.

1 Parameters estimation for HMRF model

We use the following iterative algorithm to estimate the parameters and the posterior probability of $P(I_j|\mathbf{Z})$:

1. Use model based clustering (Fraley & Raftery, 2002) to initialize the hidden states $\mathbf{I}^{(0)}$ and parameters $\widehat{\mu}^{(0)}$, $\widehat{\sigma}^{2\ (0)}$.

2. Initialize
$$\widehat{b}^{(0)} = \log\left(\sum_j \widehat{I}_j^{(0)}/n\right),$$
and $\widehat{c}^{(0)} = 0$.

3. For $t = 1, ..., T$

   (a) Update $(\widehat{b}^{(t-1)}, \widehat{c}^{(t-1)}) \to (\widehat{b}^{(t)}, \widehat{c}^{(t)})$ by maximizing the pseudo likelihood:
$$\prod_j \frac{\exp\{bI_j + cI_j\Sigma_j.\mathbf{I}\}}{\exp\{bI_j + cI_j\Sigma_j.\mathbf{I}\} + \exp\{b(1-I_j) + c(1-I_j)\Sigma_j.\mathbf{I}\}}$$
over (b, c), starting the search at $(\widehat{b}^{(t-1)}, \widehat{c}^{(t-1)})$.

   (b) Apply a single cycle of iterative conditional mode (ICM) algorithm to update $\mathbf{I}$. Specifically, we obtain a new $\widehat{I}_j^{(t)}$ based on:
$$P(I_j = 1|\mathbf{Z}; \widehat{\mathbf{I}}_{-j}, \widehat{b}^{(t)}, \widehat{c}^{(t)}) \propto f(z_j|\widehat{I}_j)P(I_j|\widehat{\mathbf{I}}; \widehat{b}^{(t)}; \widehat{c}^{(t)}).$$

   (c) Update $(\widehat{\mu}^{(t-1)}, \widehat{\sigma}^{2\ (t-1)})$ to $(\widehat{\mu}^{(t)}, \widehat{\sigma}^{2\ (t)})$:
$$\widehat{\mu}^{(t)} = \frac{\sum_j P(I_j = 1|\mathbf{Z}, \widehat{b}^{(t)}; \widehat{c}^{(t)})z_j}{\sum_j P(I_j = 1|\mathbf{Z}, \widehat{b}^{(t)}; \widehat{c}^{(t)})},$$
$$\widehat{\sigma}^{2\ (t)} = \frac{\sum_j P(I_j = 1|\mathbf{Z}, \widehat{b}^{(t)}; \widehat{c}^{(t)})(z_j - \widehat{\mu}^{(t)})^2}{\sum_j P(I_j = 1|\mathbf{Z}, \widehat{b}^{(t)}; \widehat{c}^{(t)})}.$$

4. Return $(\widehat{b}, \widehat{c}, \widehat{\mu}, \widehat{\sigma}^2) = (\widehat{b}^{(T)}, \widehat{c}^{(T)}, \widehat{\mu}^{(T)}, \widehat{\sigma}^{2\ (T)})$.

After we obtain the parameters of the Ising model $(\widehat{b}, \widehat{c}, \widehat{\mu}, \widehat{\sigma}^2)$ for each module, we then calculate the weighted average of $\widehat{\mu}$, $\widehat{\sigma}^2$ using the parameter estimates from all modules,

with weight proportional to the number of nodes per module. Two parameters ($\widehat{b}$ and $\widehat{c}$) remain module specific, and two others ($\widehat{\mu}$, $\widehat{\sigma}^2$) are common across modules. We then use the revised set of parameters to update the posterior probability for each node. Nodes which have posterior probability $P(I_j = 0|\mathbf{Z})$ less than 0.5 are selected as nASD nodes.

2 Parameters estimation for the hierarchical model

After we identify risk nodes using the HMRF algorithm, genes are further analyzed to identify risk genes (rASD genes) within the set of risk nodes identified by network analysis (nASD genes). This step is essential for all nASD genes, but it is especially important for genes within supernodes. The posterior probability a supernode is a risk node can be interpreted as the overall level of confidence that at least one gene in the supernode is a risk gene. Subsequent inference is carried out for each risk node to determine which, if any, members can be labeled as risk genes at a preset level of FDR control. To do so we compute the posterior probability for each gene within a risk node, working across all risk nodes and modules simultaneously. Consider the following hierarchical model:

$$
\begin{aligned}
Z_{jk} &\sim P(I_{jk} = 0)N(0, \sigma_0^2) + P(I_{jk} = 1)N(\mu, \sigma_1^2), \\
I_{jk} &\sim \text{Bernoulle}(\theta_j),
\end{aligned}
$$

where $j = 1, ...M$ and M is the number of nodes, and k represents gene k in the j-th node.

The parameters can be estimated by applying the EM algorithm iteratively until convergence is achieved:

$$
\begin{aligned}
\mu^{(t)} &= \frac{\sum h^{(t)}(jk) Z_{jk}}{\sum h^{(t)}(jk)}, \\
\sigma_0^{2\ (t)} &= \frac{\sum (1 - h^{(t)}(jk)) Z_{jk}^2}{\sum (1 - h^{(t)}(jk))}, \\
\sigma_1^{2\ (t)} &= \frac{\sum h^{(t)}(jk)(Z_{jk} - \mu^{(t)})^2}{\sum h^{(t)}(jk)}, \\
\theta_j^{(t)} &= \frac{\sum h^{(t)}(jk)}{n_j}.
\end{aligned}
$$

The posterior probability the j'th gene in the k'th supernode is a risk gene is

$$h^{(t)}(jk) = P(I_{jk} = 1|Z, \theta_j^{(t)}, \mu^{(t)}) = \frac{\theta_j^{(t)} P(Z_{jk}|I_{jk} = 1, \mu^{(t)})}{\theta_j^{(t)} P(Z_{jk}|I_{jk} = 1, \mu^{(t)}) + (1 - \theta_j^{(t)}) P(Z|I_{jk} = 0)} \quad (1)$$

After we obtain $P(I_{jk} = 0|Z, \theta_j^{(T)})$, the FDR is then computed to identify the significant genes within the risk nodes.

This procedure is not suitable for small supernodes ($<$ 10 genes) and single gene nodes because there is not sufficient information from which to estimate θ_j for each small node. Thus we adjust the algorithm using a two-stage procedure. First, for those nASD genes that fall into large supernodes ($\geq$ 10 genes), the proportion of risk genes in supernode j, θ_j, is estimated for each individual supernode, but the mean and standard deviation μ and σ are estimated jointly over all nASD genes in the big supernodes. Thus using (θ_j, μ, σ) in Eq. (1) we compute the posterior probability for each gene in each large supernode. Next, we compute θ as the average of the θ_j's estimated from big supernodes. Using (θ, μ, σ) in Eq. (1) we compute the posterior probability for the remaining nASD genes. Finally, let $q_{(s)}$ be the sorted posterior probability in descending order, the Bayesian FDR correction (Müller et al., 2006) of the l'th sorted gene can be calculated as

$$\text{FDR}_l = \sum_{s=1}^{l} q_{(s)}/l$$

Genes with FDR less than 0.05 are selected as the rASD genes.

References

Benjamini, Y., & Hochberg, Y. (1995). Controlling the false discovery rate: a practical and powerful approach to multiple testing. *J R Stat Soc Series B Stat Methodol.*, (pp. 289–300).

Fraley, C., & Raftery, A. (2002). Model-based clustering, discriminant analysis, and density estimation. *J Am Stat Assoc.*, *97*(458), 611–631.

He, X., Sanders, S. J., Liu, L., et al. (2013). Integrated model of de novo and inherited genetic variants yields greater power to identify risk genes. *PLoS genetics*, *9*(8), e1003671.

Langfelder, P., & Horvath, S. (2008). WGCNA: an R package for weighted correlation network analysis. *BMC bioinformatics*, *9*(599).

Müller, P., Parmigiani, G., & Rice, K. (2006). FDR and Bayesian multiple comparisons rules. *Bayesian Statistics*, *8*, 349–470.
