# Supplementary material for: DAWN: a framework to identify autism genes and subnetworks using gene expression and genetics
Source: Mol Autism. 2014 Mar 6;5:22. doi: 10.1186/2040-2392-5-22 (PMC4016412; doi:10.1186/2040-2392-5-22)
Supplement: Additional file 11 — Table S5. Summary of de novo variants identified for 44 selected genes for the MIPS experiment. [file 2040-2392-5-22-S11.docx]

Table S5

| *De novo* mutation  in gene | | Count  in genes | |
| --- | --- | --- | --- |
| Prior  sample | New sample  (N=2448) | rASD | Not  rASD |
| Yes | Yes | 4 | 1 |
|  | No | 4 | 7 |
| No | Yes | 2 | 1 |
|  | No | 0 | 25 |
