# Supplementary material for: DAWN: a framework to identify autism genes and subnetworks using gene expression and genetics
Source: Mol Autism. 2014 Mar 6;5:22. doi: 10.1186/2040-2392-5-22 (PMC4016412; doi:10.1186/2040-2392-5-22)
Supplement: Additional file 13 — Figure S7. Enrichment analysis using genes from the clusters shown in Figure 4 with the ChEA, Wikipathways, GO_biological Process, MGI_Mouse Phenotype and Human Gene Atlas gene-set libraries. [file 2040-2392-5-22-S13.pdf]

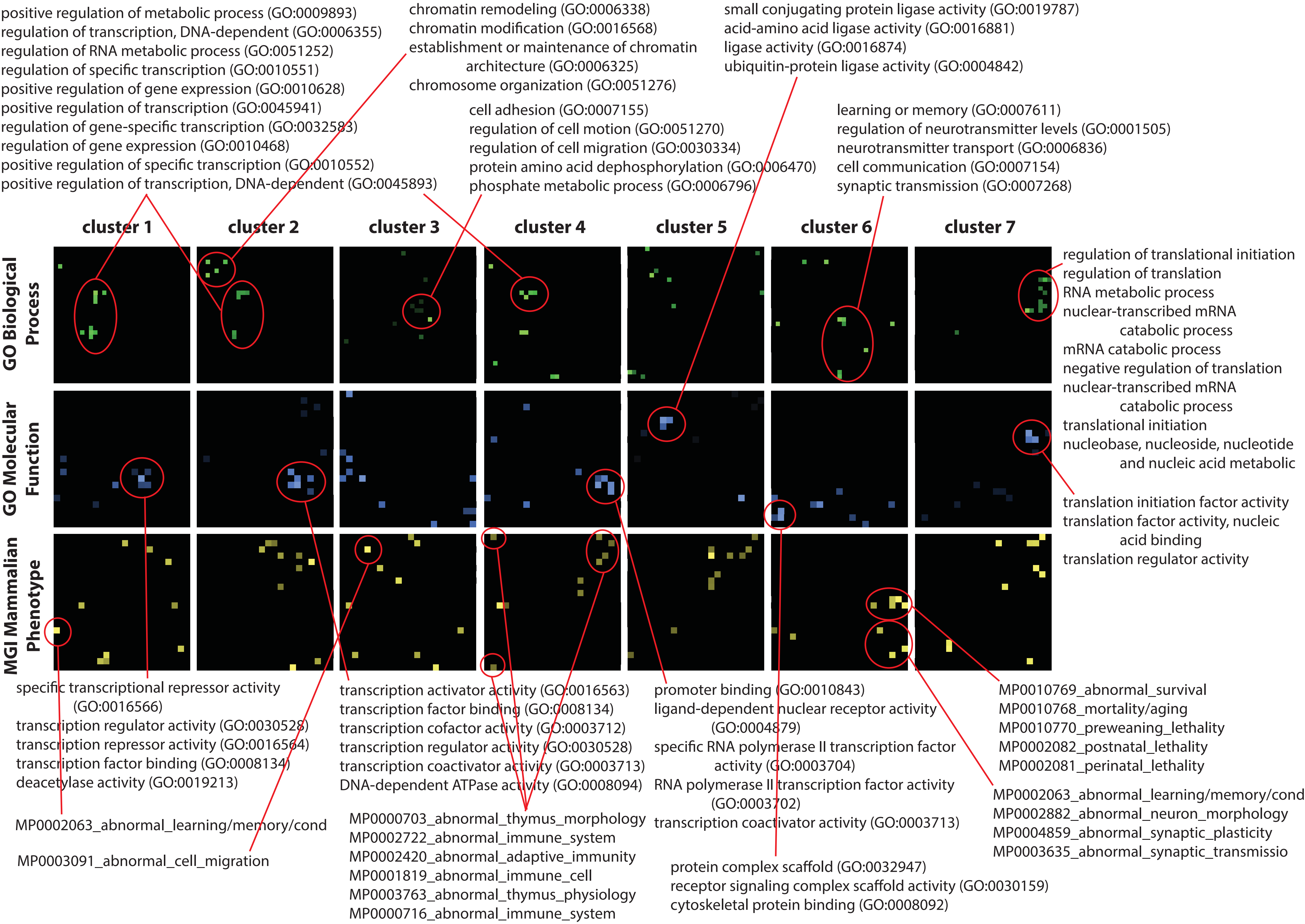
positive regulation of metabolic process (GO:0009893)
regulation of transcription, DNA-dependent (GO:0006355)
regulation of RNA metabolic process (GO:0051252)
regulation of specific transcription (GO:0010551)
positive regulation of gene expression (GO:0010628)
positive regulation of transcription (GO:0045941)
regulation of gene-specific transcription (GO:0032583)
regulation of gene expression (GO:0010468)
positive regulation of specific transcription (GO:0010552)
positive regulation of transcription, DNA-dependent (GO:0045893)
chromatin remodeling (GO:0006338)
chromatin modification (GO:0016568)
establishment or maintenance of chromatin architecture (GO:0006325)
chromosome organization (GO:0051276)
cell adhesion (GO:0007155)
regulation of cell motion (GO:0051270)
regulation of cell migration (GO:0030334)
protein amino acid dephosphorylation (GO:0006470)
phosphate metabolic process (GO:0006796)
small conjugating protein ligase activity (GO:0019787)
acid-amino acid ligase activity (GO:0016881)
ligase activity (GO:0016874)
ubiquitin-protein ligase activity (GO:0004842)
learning or memory (GO:0007611)
regulation of neurotransmitter levels (GO:0001505)
neurotransmitter transport (GO:0006836)
cell communication (GO:0007154)
synaptic transmission (GO:0007268)
cluster 1
cluster 2
cluster 3
cluster 4
cluster 5
cluster 6
cluster 7
GO Biological Process
GO Molecular Function
MGI Mammalian Phenotype
regulation of translational initiation
regulation of translation
RNA metabolic process
nuclear-transcribed mRNA catabolic process
mRNA catabolic process
negative regulation of translation
nuclear-transcribed mRNA catabolic process
translational initiation
nucleobase, nucleoside, nucleotide and nucleic acid metabolic
translation initiation factor activity
translation factor activity, nucleic acid binding
translation regulator activity
specific transcriptional repressor activity (GO:0016566)
transcription regulator activity (GO:0030528)
transcription repressor activity (GO:0016564)
transcription factor binding (GO:0008134)
deacetylase activity (GO:0019213)
MP0002063_abnormal_learning/memory/cond
MP0003091_abnormal_cell_migration
transcription activator activity (GO:0016563)
transcription factor binding (GO:0008134)
transcription cofactor activity (GO:0003712)
transcription regulator activity (GO:0030528)
transcription coactivator activity (GO:0003713)
DNA-dependent ATPase activity (GO:0008094)
MP0000703_abnormal_thymus_morphology
MP0002722_abnormal_immune_system
MP0002420_abnormal_adaptive_immunity
MP0001819_abnormal_immune_cell
MP0003763_abnormal_thymus_physiology
MP0000716_abnormal_immune_system
promoter binding (GO:0010843)
ligand-dependent nuclear receptor activity (GO:0004879)
specific RNA polymerase II transcription factor activity (GO:0003704)
RNA polymerase II transcription factor activity (GO:0003702)
transcription coactivator activity (GO:0003713)
protein complex scaffold (GO:0032947)
receptor signaling complex scaffold activity (GO:0030159)
cytoskeletal protein binding (GO:0008092)
MP0010769_abnormal_survival
MP0010768_mortality/aging
MP0010770_preweaning_lethality
MP0002082_postnatal_lethality
MP0002081_perinatal_lethality
MP0002063_abnormal_learning/memory/cond
MP0002882_abnormal_neuron_morphology
MP0004859_abnormal_synaptic_plasticity
MP0003635_abnormal_synaptic_transmissio
