# Supplementary material for: DAWN: a framework to identify autism genes and subnetworks using gene expression and genetics
Source: Mol Autism. 2014 Mar 6;5:22. doi: 10.1186/2040-2392-5-22 (PMC4016412; doi:10.1186/2040-2392-5-22)
Supplement: Additional file 16 — Table S7. Correlations amongst neurexin and neuroligin genes for periods 4–6 (top) and 3–5 (bottom). [file 2040-2392-5-22-S16.docx]

Table S7. Correlations amongst neurexin and neuroligin genes for the periods 4-6 (top) and periods 3-5 (bottom).

| Gene | *NRXN1* | *NRXN2* | *NRXN3* | *NLGN1* | *NLGN2* | *NLGN3* | *NLGN4X* |
| --- | --- | --- | --- | --- | --- | --- | --- |
| *NRXN1* |  | 0.63 | 0.42 | 0.20 | 0.26 | 0.52 | 0.62 |
| *NRXN2* | 0.48 |  | 0.42 | 0.39 | 0.56 | 0.54 | 0.50 |
| *NRXN3* | 0.31 | 0.07 |  | 0.54 | 0.07 | 0.24 | 0.57 |
| *NLGN1* | 0.09 | 0.15 | 0.43 |  | 0.29 | 0.06 | 0.70 |
| *NLGN2* | 0.10 | 0.56 | 0.13 | 0.20 |  | 0.39 | 0.17 |
| *NLGN3* | 0.07 | 0.22 | 0.21 | 0.59 | 0.20 |  | 0.12 |
| *NLGN4X* | 0.39 | 0.20 | 0.39 | 0.74 | 0.12 | 0.47 |  |
